# In Vitro and In Silico Anti-Acetylcholinesterase Activity from *Macaranga tanarius* and *Syzygium jambos*

**DOI:** 10.3390/molecules27092648

**Published:** 2022-04-20

**Authors:** Mira Syahfriena Amir Rawa, Nurul Amira Nurul Azman, Suriani Mohamad, Toshihiko Nogawa, Habibah A. Wahab

**Affiliations:** 1 Collaborative Laboratory for Herbal Standardization (CHEST), School of Pharmaceutical Sciences, Universiti Sains Malaysia, George Town 11800, Penang, Malaysia; mirasyah@usm.my; 2 Chemical Biology Research Group, RIKEN Center for Sustainable Resource Science, 2-1 Hirosawa, Wako 351-0198, Saitama, Japan; 3 USM-RIKEN Interdisciplinary Collaboration for Advanced Sciences (URICAS), Universiti Sains Malaysia, George Town 11800, Penang, Malaysia; nurulamira.nazman@gmail.com (N.A.N.A.); suriani@usm.my (S.M.)

**Keywords:** *Macaranga tanarius*, *Syzygium jambos*, acetylcholinesterase inhibitor, prenylflavonoid, anacardic acid, isolation, structure elucidation, in vitro, in silico

## Abstract

*Macaranga tanarius* (MT) and *Syzygium jambos* (SJ) are pharmacologically reported to have anti-oxidant, anti-inflammatory, and anti-diabetic effects, and can be neuroprotective agents. Our previous work revealed that MT and SJ exhibited 76.32% and 93.81% inhibition against acetylcholinesterase (AChE) at 50 μg/mL final concentration in their ethyl acetate and hexane fractions, respectively. This study was aimed to investigate the bioactive constituents of MT and SJ and their molecular mechanism toward AChE inhibition. Bioassay-guided isolation afforded prenylflavonoids **1**–**3** from MT and anacardic acid derivatives **4** and **5** from SJ that were confirmed by NMR and MS data. Compound **5** exerted the strongest anti-AChE potential (IC_50_: 0.54 μM), followed by **1**, **4**, **3**, and **2** (IC_50_: 1.0, 2.4, 6.8, and 33 μM, respectively). In silico molecular docking revealed **5** formed stronger molecular interactions including three H-bonds than its derivative **4** based on the saturation of their alkyl chains. The addition of a five carbon-prenyl chain in **1** increased the number of binding interactions, justifying its greater activity than derivatives **2** and **3**. This research reflects the first report of AChE inhibitors from these species, thereby adding pharmacological values to MT and SJ as potential remedies in neuroprotection.

## 1. Introduction

*Macaranga tanarius* (L.) Mull.Arg. (MT), from the Euphorbiaceae family is a fast-growing evergreen tree that can reach a height up to 20 m [1]. It is native to Malaysia as well as other tropical regions such as Africa, Madagascar, Southeast Asia, Australia, and the Pacific. MT traditionally has been used in folk medicine as anti-pyretic and anti-tussive agents to relieve fever and cough [2]. The anti-inflammatory properties of its leaves are used to treat wounds and swellings [2,3]. *Macaranga* species are rich in flavonoids and stilbenes, which are thought to be responsible for a variety of pharmacological activities such as anti-cancer, anti-oxidant, anti-microbial, anti-inflammatory, and anti-diabetic properties [4,5,6,7].

*Syzygium jambos* (L.) Alston (SJ), also known as rose apple is widely distributed in Central America, sub-Saharan Africa, and Asia, including Malaysia [8]. It is a member of the Myrtaceae family and has long been used in folk medicine for its anti-pyretic and anti-inflammatory properties. The leaves are decocted for diuretic, rheumatism, and sore eyes relief, while the seeds are used to treat diarrhea, dysentery, diabetes, and catarrh [8,9]. The bark extract is used to soothe asthma and bronchitis [8,9]. SJ was reported to comprise primarily gallic acid, vitamin C, cyanidins, tannins, and anthocyanins that contribute to its anti-diabetic, anti-oxidant, and anti-inflammatory properties [10,11,12].

Our ongoing search for acetylcholinesterase (AChE) inhibitors has been focused on Malaysian plants exhibiting anti-inflammatory, anti-oxidant, and anti-diabetic activities that are considered as neuroprotective agents [13]. AChE is crucial in both central and peripheral nervous systems, as it degrades the neurotransmitter acetylcholine homeostasis [14]. However, in patients inflicted with Alzheimer’s disease, the progression of this illness is associated with low levels of acetylcholine, possibly due to increased hydrolysis of acetylcholine molecules by AChE [14]. Therefore, to achieve a homeostatic neurotransmitter equilibrium, AChE inhibitors play a critical role in preventing the AChE activity from breaking down more neurotransmitters. Physostigmine and tacrine are among the early discovered drugs used to inhibit cholinesterase, while rivastigmine, galanthamine, and donepezil are the currently approved AChE inhibitors [15].

In our previous report, MT and SJ leaves revealed strong AChE inhibition in the ethyl acetate (76.32%) and hexane (93.81%) extracts, respectively, at 50 μg/mL final concentration (Supplementary Materials Figure S1) [13]. As both species were reported to promote anti-inflammatory, anti-oxidant, and anti-diabetic properties, their anti-AChE potential has yet to be addressed; this study was initiated to isolate the active principles from these extracts by anti-AChE assay-guided chromatography techniques. The structures were identified on the basis of NMR and MS spectral data, and the enzyme–ligand interactions were predicted based on Autodock 4.3 molecular docking.

## 2. Results and Discussion

### 2.1. Isolation and Identification of Active Constituents

Our previous screening study evaluated 177 Malaysian plant extracts for their anti-AChE potential [13]. Among the 18 plant extracts reported to show strong anti-cholinesterase activity at 50 μg/mL final concentration, *Artocarpus altilis* (AA) and MT exhibited more pronounced inhibitory activity in the ethyl acetate fraction, while SJ exhibited stronger activity in the hexane fraction (Figure S1). Identification of AA’s bioactive principles was hampered by the low amount of sample collected and, therefore, was not pursued. Moreover, the bioactive principles from butanol and water fractions constitute high molecular weight polyphenols [13]. Our interest to identify low molecular weight active constituents, which have not been reported from MT and SJ before, led to this bioassay-guided isolation.

About 991 mg of ethyl acetate fraction from MT leaves was chromatographed by MPLC to obtain 12 sub-fractions (F0001-F0012) (Figure S2). Further MPLC on Active F0002 afforded crude **1** and **2** sub-fractions. Purification of crude **1** fraction using ODS-MPLC (isocratic 85% acetonitrile) harbored 31.4 mg of compound **1**. Purification of crude **2** using ODS-HPLC, isocratic 60% acetonitrile, yielded 3.6 mg of **2**. Active F0008 was also chromatographed by ODS-MPLC (isocratic 60% acetonitrile) to obtain a crude compound **3**, which was purified by ODS-HPLC (isocratic 90% acetonitrile) to give 2.7 mg of **3**. About 5 g hexane fraction of SJ leaves was separated by MPLC with a stepwise solvent gradient to obtain 8 sub-fractions (F0001–F008) (Figure S3). Active F0002 was further separated by MPLC and open column chromatography packed with Sephadex LH-20 using a ratio of 4:5:1 hexane:ethyl acetate:methanol solvent system to afford compounds **4** and **5** rich-fraction. Purification of the active constituents were achieved by ODS-HPLC (water/acetonitrile) to yield **4** (27.0 mg) and **5** (78.1 mg).

The structures of isolated compounds (Figure 1) were identified based on 1D and 2D NMR and MS spectral data (Figures S4–S34). Their physicochemical properties including ^1^H and ^13^C NMR chemical shifts were compared and identical to the reported data (Tables S1–S6). Compounds **1**, **2,** and **3** from MT were deduced to have a flavanone backbone structure based on their UPLC-MS analysis revealing λ_max_ around 233, 290, and 334 nm (Figure S35). Compounds **4** and **5** from SJ showed UV characteristics of λ_max_ at 243 and 311 nm (Figure S36).

Compound **1** was obtained as a light yellow amorphous solid. The ^1^H NMR spectrum of **1** showed three singlet methyl signals (δ_H_ = 1.54, 1.60, and 1.75), a doublet signal integrating two methyl protons (δ_H_ = 1.65), one methylene α signal to the carbonyl (δ_H_ = 2.59, dd and 3.09, dd), as well as seven methine signals above 5.00 ppm (Table S2). The observation of four methylene signals suggested the presence of side chains. Its ^13^C NMR indicated 30 carbon signals including a carbonyl signal (δ_C_ = 198.4) and one oxymethine signal (δ_C_ = 77.9). These data were in concordant with the MS spectrum of **1**. Its ESI-MS revealed quasi-molecular ions observed at m/z 493.8 [M+H]^+^ and 491.9 [M-H]^-^ (Figure S30), which suggested a formula of C_30_H_36_O_6_. The 2D NMR correlations suggested **1** had a flavanone skeleton with a B-ring substitution pattern. The presence of a geranyl chain and a prenyl chain were observed, where the methylene (δ_H_ = 3.21, d) was correlated to A-ring carbons (δ_C_ = 166.1, 109.8, and 162.6) and the methylene (δ_H_ = 3.47, d) was correlated to B-ring carbons (δ_C_ = 128.3 and 144.5). These characteristics proposed **1** was either nymphaeol C or solophenol A. It is evident that proton H-1′’’ was correlated to carbon C-7, C-8, and C-9, and proton H-6 was correlated to carbon C-5, C-7, C-8, and C-10, confirming the position of the geranyl chain that was attached to C-8. Compound **1** hence was identified as solophenol A. It was first reported in propolis collected from the Solomon Islands [16].

Compound **2** showed a similar characteristic of geranylation at C-2′ on ring B to **1,** but lacked a prenyl chain on ring A. Its ESI-MS spectrum showed quasi-molecular ions at m/z 425.8 [M+H]^+^ and 423.3 [M-H]^−^ (Figure S31). These characteristics indicated **2** was nymphaeol B, a known compound identified in a few *Macaranga* species [4]. The absence of ortho-coupling in **3** suggested it had no vicinal protons in ring B. Based on HSQC correlations of **3**, the singlet proton (δ_H_ = 6.79) integrating a total of two hydrogen atoms was assigned to C-2′ and C-4′, while the singlet proton (δ_H_ = 6.91) was assigned to C-6′ (Table S3). The doublet proton H-1” expressing HMBC correlations with C-5, C-6, and C-7 indicated that geranylation occurred at C-6 on ring A. Compound **3** showed similar MS characteristics as **2**, in having a geranyl chain (Figure S32 and Table S4), and therefore was identified as schizolaenone C. It was first isolated from *Schizolaena hystrix* [17]. Compounds **1** and **3** were newly identified from Macaranga species.

The NMR spectra of **4** and **5** proposed the presence of a carboxyl, hydroxyl, and long chain of methylene groups attached to an aromatic ring (Tables S5 and S6). It is apparent from their structures that the presence of 1,2,3-trisubstituted phenyl moiety represented 6-alkysalicylic acid, an anacardic acid derivative. Six olefinic protons from **4** and four olefinic protons from **5** showed HMBC correlations in between the long aliphatic chain, indicating three double bonds and two double bonds were present at their aliphatic chain, respectively. Quasi-molecular ions of m/z 371 [M+H]^+^ and 369 [M-H]^-^ were observed in **4** (Figure S33), while **5** showed m/z 373 [M+H]^+^ and 371 [M-H]^-^ in the MS spectrum (Figure S34). Compound **4** was identified as 6-heptadeca-8Z,11Z,14Z-trienyl salicylic acid (SB-202742) [18] and **5** was identified as 6-heptadeca-9Z,12Z-dienyl salicylic acid (anacardic acid C) [19]. Both compounds were reported for the first time in SJ.

### 2.2. In Vitro Anti-AChE Activity

Compound **1** manifested the lowest IC_50_ value against AChE at 1.0 μM among the prenylflavonoids, followed by **3** and **2**, which showed moderate to low AChE inhibition (Table 1). The different position of geranyl group appeared to influence the activity of **2** and **3**, where geranylation on ring A in **3** might contribute to a better inhibition than on ring B. However, **1** showed greater activity by seven times than **3**, which could be postulated due to the presence of a prenyl group in **1**. On the other hand, **5** demonstrated the strongest anti-AChE activity among all isolated compounds with an IC_50_ value of 0.5 μM (Table 1). Anacardic acid derivatives are the major constituents of cashew nutshell liquid (CNSL), whereby different degrees of saturation were found on the side chains [20]. It was suggested that the saturation level found on their aliphatic chain could affect biological activities. Compound **4,** which contained an extra double bond on the aliphatic chain, exhibited a weaker AChE inhibition by four times than **5**. Higher saturated anacardic acids such as **5** seemed to have a flexible structural conformation during enzyme inhibition, but further molecular docking is necessary to confirm.

### 2.3. In Silico Molecular Docking

Compounds **1**–**3** showed binding energies around −10.5 to −12.6 kcal/mol, while the binding energies of **4** and **5** fell within the range −7.0 to −8.0 kcal/mol, implying ideal values for the formation of a stable complex with the target enzyme were observed (Table 2). Their varying molecular weights may explain the differences in their binding energy values.

**Figure 2:**
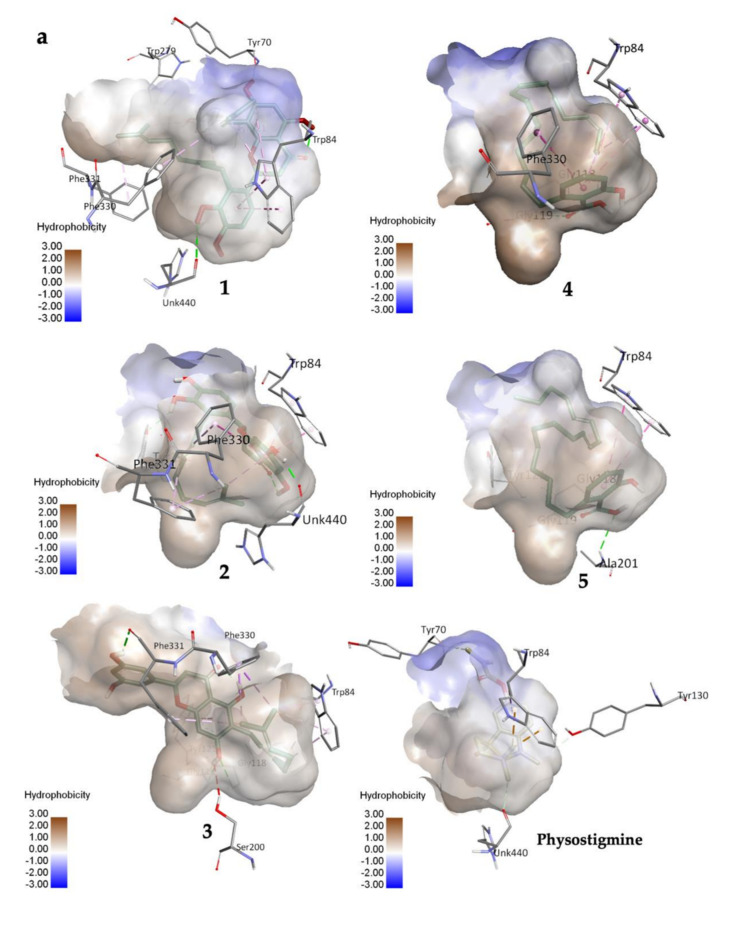

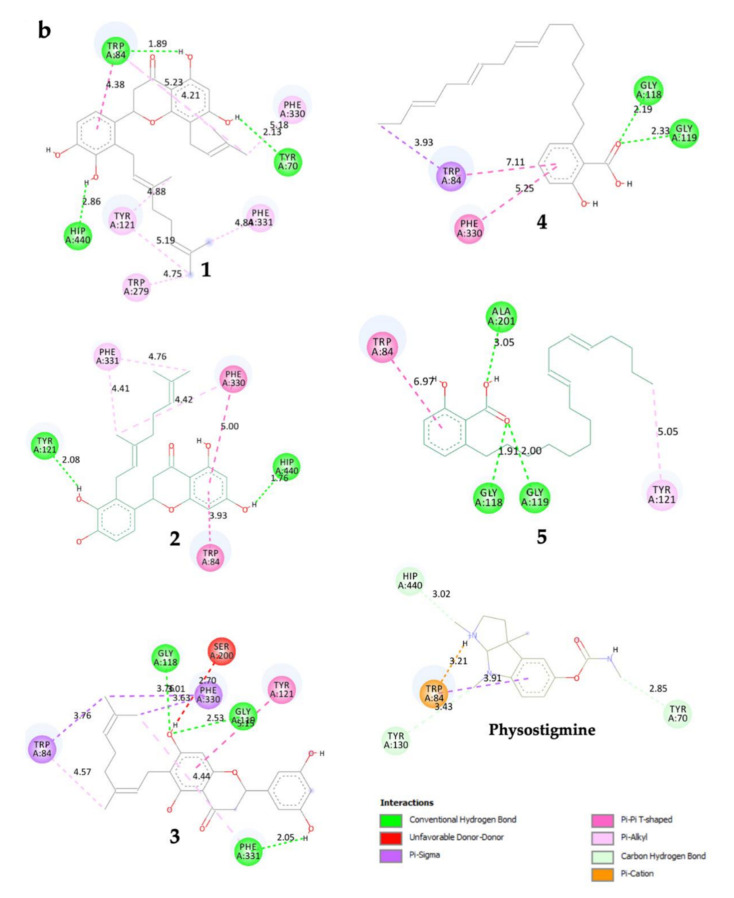
(**a**) 3D representation of molecular interactions between **1** and **5** and TcAChE residues (PDB ID: 1W6R) (**b**) 2D representation of molecular interactions between **1** and **5** and TcAChE residues.

Compound **1** established three polar interactions with TYR 70, TRP 84, and HIS 440, and seven non-polar interactions with TRP 84, TYR 121, TRP 279, PHE 330, and PHE 331 (Figure 2a; Table 2). In addition, all compounds **1**–**3** interacted with four similar key TcAChE residues: TRP 84, TYR 121, PHE 330, and PHE 331 (Figure 2a; Table 2). Compound **2** revealed seven interactions including two conventional H-bonds. On the other hand, **3** showed eight interactions including three H-bonds, and hence justified its superior inhibitory activity than **2**. It is worth mentioning that **1** had shorter distances of H-bonds than **3**. In addition, the prenyl group of **1** formed a few contacts with TcAChE residues, and therefore may help in creating stronger molecular interactions with the enzyme.

Compounds **4** and **5** formed three similar interactions with TRP 84 (non-polar), GLY 118, and GLY 119 (Figure 2b; Table 2). However, **5** made one extra polar H-bond with ALA 201 and a π–alkyl non-polar interaction with TYR 121. Its saturated alkyl chain was observed to create more flexible conformation than **4**, allowing more interactions especially H-bonds with the enzyme residues. Anacardic acid derivatives formed interactions mostly at the oxyanion hole (OH), which is located near the base cavity. Conversely, prenylflavonoids favored binding interactions at the peripheral active site (PAS) located near the gorge mouth, where the entrance was blocked. Further molecular dynamics study is necessary, but these results rationalized their in vitro activity.

## 3. Materials and Methods

### 3.1. Chemicals and Instruments

In vitro enzyme assay reagents were purchased as previously described [13]. Acetylcholinesterase (AChE) from *Electrophorus electricus* type VI-S lyophilized powder (137 units/mg) was acquired from Sigma Aldrich (St. Louis, MO, USA). Substrate acetylthiocholine iodide (ATCl) and sodium phosphate monobasic and dibasic anhydrous used for buffer preparation were obtained from Wako Pure Chemical (Osaka, Japan). Coloring reagent 5,5′-dithio-bis-(2-nitrobenzoic acid) (DTNB) was acquired from Nacalai Tesque (Kyoto, Japan). Positive control physostigmine was obtained from TCI Tokyo Chemical Industry (Tokyo, Japan). All other reagents acquired from commercial sources were of analytical grade.

Hits Biomicroplate reader (Scinics, Tokyo, Japan) was used to measure absorbance. Buchi R-215 Rotavapor (Buchi, Flawil, Switzerland) was used for extraction. Waters UPLC-H-Class system (Waters, Milford, MA, USA) connected to AB Sciex API 3200 by ESI probe (AB Sciex, Framingham, MA, USA) on a Waters BEH C18 column (2.1 mm i.d. × 50 mm, 1.7 μm) was used to perform LC-MS analysis with elution of acetonitrile/0.05% aqueous formic acid linear gradient system (acetonitrile: 5 to 100% in 4 min at 0.5 mL min^−1^). To obtain data at 500 MHz for ^1^H NMR and 125 MHz for ^13^C NMR, JEOL JNM-ECA-500 spectrometer (JEOL, Tokyo, Japan) was run, where chemical shifts (in ppm) were referenced based on the residual undeuterated solvent. Isolation was performed using MPLC on Teredyne ISCO CombiFlash Companion (Teredyne ISCO, Lincoln, NE, USA) and RediSep *Rf* Gold silica column or RediSep *Rf* Gold HP C18. Purification was achieved through preparative HPLC using Waters 600E pump system with Senshu Pak Pegasil ODS column (20 mm i.d. × 250 mm or 10 mm i.d. × 250 mm, 5 μm).

### 3.2. Plant Materials

*Macaranga tanarius* (L.) Mull.Arg. (MT) and *Syzygium jambos* (L.) Alston (SJ) leaves were collected in Raub, Pahang, Malaysia. The species were authenticated by the Forest Research Institute Malaysia (FRIM), Kuala Lumpur with specimen IDs: PID 030120-02 (MT) and PID 020120-02 (SJ). The plant names were checked and confirmed from the website “The Plant List” (www.theplantlist.org, accessed on 7 February 2020).

### 3.3. Plant Extraction and Partition

General plant extraction and partition were carried out according to the method previously described [13]. Four fractions, hexane (487 mg), ethyl acetate (991 mg), butanol (150 mg), and water (370 mg) were obtained from the extraction of MT leaves (40 g). Extraction and partition of SJ leaves (1.36 kg) resulted in four fractions, hexane (10 g), ethyl acetate (18 g), butanol (21 g), and water (82 g).

### 3.4. Isolation of Bioactive Constituents

The active ethyl acetate fraction of MT leaves (991 mg) was chromatographed by MPLC with a stepwise solvent gradient (chloroform/methanol) to obtain 12 sub-fractions (F0001-F0012) (Figure S2). Active F0002 (190 mg) was further separated by MPLC with a stepwise gradient solvent system (hexane/ethyl acetate) to obtain crude **1** and **2** sub-fractions. Further purification of crude **1** fraction (66.2 mg) using ODS-MPLC (isocratic 85% acetonitrile) harbored 31.4 mg of **1**. Purification of crude **2** (19.2 mg) using ODS-HPLC, isocratic 60% acetonitrile, yielded 3.6 mg of **2**. Active F0008 (65.6 mg) was also chromatographed by ODS-MPLC (isocratic 60% acetonitrile) to obtain a crude compound **3**, which was purified by ODS-HPLC (isocratic 90% acetonitrile) to give 2.7 mg of **3**.

The active hexane fraction of SJ leaves (5 g) was separated by MPLC with a stepwise solvent gradient (hexane/acetone) to obtain 8 sub-fractions (F0001-F008) (Figure S3). Active F0002 (1.6 g) was MPLC chromatographed using a step-wise gradient solvent system (hexane/ethyl acetate) in which harbored active F0014 sub-fraction (371.3 mg). Further separation of F0014 by open column chromatography packed with Sephadex LH-20 was performed using a ratio of 4:5:1 hexane:ethyl acetate:methanol solvent system to afford compounds **4** and **5** rich-fraction (185.1 mg). Purification of the active constituents were achieved by ODS-HPLC (water/acetonitrile) to yield **4** (27.0 mg) and **5** (78.1 mg).

### 3.5. In Vitro Anti-Acetylcholinesterase Assay

The plant fractions and isolated compounds were evaluated for their anti-AChE potential in triplicate based on the Ellman’s method described previously [13]. Physostigmine was used as a positive control.

### 3.6. In Silico Molecular Docking

The crystal structure of *Torpedo californica* acetylcholinesterase (TcAChE) in complex with galanthamine [21] (PDB: 1W6R, 2.05 Å) was retrieved from the Research Collaboratory for Structural Bioinformatics (RCSB) Protein Data Bank (www.rcsb.org, accessed on 24 November 2021). TcAChE was selected in this study based on literature reviews [22,23], where it depicts closely the in vitro model. The water molecules and heteroatoms were removed using UCSF Chimera version 1.15. To add hydrogens, reconstruct missing atoms, and assign atomic charges, the PDB2PQR (https://server.poissonboltzmann.org/pdb2pqr, accessed on 24 November 2021) and MolProbity (http://molprobity.biochem.duke.edu, accessed on 24 November 2021) web services were used. The protonation state for the ionizable groups of the protein was set at 7.40 by using the most used empirical pKa predictor (PROPKA3). The protein was then added with hydrogen atoms and Kollman charges using AutoDock Tools 1.5.6. The 3D ligand structures were acquired from PubChem (https://pubchem.ncbi.nlm.nih.gov, accessed on 24 November 2021), structurally minimized, and added with Gaisteger charges using UCSF Chimera. The ligand structure then was prepared with torsion using AutoDock Tools 1.5.6. A control docking using galanthamine (heteroatom GNT) was conducted, whereby the docking grid was set to 50, 50, and 50 of X-, Y-, and Z-dimensions with 0.375 Å grid point spacing, respectively. As a result, the coordinates of central grid points were set at x = 3.518, y = 65.122, z = 64.481. The protein–ligand docking simulation was performed using AutoDock 4.2, allowing 150 confirmations of genetic algorithm (GA) run with Lamarckian GA output. The docking simulation generating the lowest free energy of binding (FEB) with the highest cluster was selected and visualized using Discovery Studio Visualizer. The molecular interactions were observed and discussed based on our comparisons with the literature reviews [22,24].

## 4. Conclusions

This study highlights the pharmacological relevance of MT and SJ as AChE inhibitors in neuroprotection. Their first report of anti-AChE activity was represented here. Compound **5** exerted the strongest anti-AChE activity at 0.54 μM of IC_50_. The in silico molecular docking supported the in vitro data, where prenylation in prenylflavonoids and saturation of the alkyl chain in anacardic acid derivatives may modulate the molecular interactions with the enzyme.

## Figures and Tables

**Figure 1:**
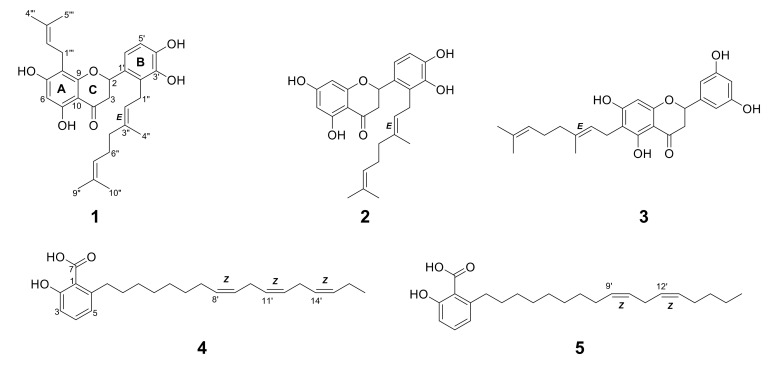
Structures of **1**–**3** from *Macaranga tanarius* and **4**–**5** from *Syzygium jambos*.

**Table 1:** IC_50_ of **1**–**5** against AChE in μM.

Compound	AChE Inhibition (μM)
**1**	1.0
**2**	33
**3**	6.8
**4**	2.4
**5**	0.54
Physostigmine	0.10

**Table 2:** Protein–ligand interactions of **1**–**5** with TcAChE (PDB ID: 1W6R).

Ligand	Free Energy Binding (FEB) in kcal/mol	Type of TcAChE Active Site	TcAChE Key Residue	Type of Binding Interaction (Distance in Å)
**1**	−12.58	PAS	TYR 70	H-bond (2.13)
		CAS	TRP 84	H-bond (1.89)
				π–π stacked (4.38)
				π–alkyl (4.21)
		PAS	TYR 121	π–alkyl (4.88, 5.19)
		PAS	TRP 279	π–alkyl (4.75)
		PAS	PHE 330	π–alkyl (5.18)
		PAS	PHE 331	π–alkyl (4.84)
		ES	HIS 440	H–bond (2.86)
**2**	−10.60	CAS	TRP 84	π–π stacked (3.93)
		PAS	TYR 121	H-bond (2.08)
		PAS	PHE 330	π–π T-shaped (5.00)
				π–alkyl (4.42)
		PAS	PHE 331	π–alkyl (4.41, 4.76)
		ES	HIS 440	H-bond (1.76)
**3**	−10.52	CAS	TRP 84	π–σ (3.76)
				π–alkyl (4.57)
				H-bond (3.75)
		OH	GLY 118	H-bond (2.53)
		OH	GLY 119	π–π T-shaped (5.15)
		PAS	TYR 121	π–σ (3.01, 3.63)
		PAS	PHE 330	H-bond (2.05)
		PAS	PHE 331	π–alkyl (4.44)
**4**	−7.94	CAS	TRP 84	π–σ (3.93)
				π–π T (7.11)
		OH	GLY 118	H-bond (2.19)
		OH	GLY 119	H-bond (2.33)
		PAS	PHE 330	π–π T-shaped (5.25)
**5**	−7.25	CAS	TRP 84	π–π T (6.97)
		OH	GLY 118	H-bond (1.91)
		OH	GLY 119	H-bond (2.00)
		PAS	TYR 121	π–alkyl (5.05)
		OH	ALA 201	H-bond (3.05)
Physostigmine (+control)	−8.81	PAS	TYR 70	C–H-bond (2.85)
		CAS	TRP 84	π–cation (3.21)
				π–σ (3.91)
		CAS	TYR 130	C–H-bond (3.43)
		ES	HIS 440	C–H-bond (3.02)
Galanthamine (redocked ligand)	−8.68 (reference RMSD: 0.71)	CAS	TRP 84	π–alkyl (5.05, 5.48)
		ES	SER 200	H-bond (2.19)
				C–H-bond (3.15)
		OH	GLY 118	H-bond (2.58)
		PAS	ASP 72	C–H-bond (3.77)
		PAS	PHE 330	π–alkyl (4.67)
		PAS	PHE 331	π–π T-shaped (5.30)

PAS: Peripheral active site, CAS: Choline ‘anionic’ site, ES: Esteratic site, OH: Oxyanion hole.

## Data Availability

The data presented in this study are available in the Supplementary Materials.

## Supplementary Materials

The following are available online at https://www.mdpi.com/article/10.3390/molecules27092648/s1, Figure S1: Anti-acetylcholinesterase (AChE) activity of 18 potent Malaysian plant fractions (hexane, ethyl acetate, butanol, and water) at 50 µg/mL final concentration; Figure S2. Isolation scheme of *Macaranga tanarius* ethyl acetate extract; Figure S3. Isolation scheme of *Syzygium jambos* hexane extract; Figure S4. ^1^H NMR spectrum of **1**; Figure S5. 1H NMR spectrum of **2**; Figure S6. ^1^H NMR spectrum of **3**; Figure S7. ^1^H NMR spectrum of **4**; Figure S8. ^1^H NMR spectrum of **5**; Figure S9. ^13^C NMR spectrum of **1**; Figure S10. ^13^C NMR spectrum of **2**; Figure S11. ^13^C NMR spectrum of **3**; Figure S12. ^13^C NMR spectrum of **4**; Figure S13. ^13^C NMR spectrum of **5**; Figure S14. DEPT135 spectrum of **1**; Figure S15. DEPT135 spectrum of **3**; Figure S16. DEPT135 spectrum of **4**; Figure S17. DEPT135 spectrum of **5**; Figure S18. HSQC spectrum of **1**; Figure S19. HSQC spectrum of **2**; Figure S20. HSQC spectrum of **3**; Figure S21. HSQC spectrum of **4**; Figure S22. HSQC spectrum of **5**; Figure S23. DQF-COSY spectrum of **1**; Figure S24. DQF-COSY spectrum of **3**; Figure S25. DQF-COSY spectrum of **5**; Figure S26. HMBC spectrum of **1**; Figure S27. HMBC spectrum of **3**; Figure S28. HMBC spectrum of **4**; Figure S29. HMBC spectrum of **5**; Figure S30. ESI-MS spectrum of **1** by UPLC-MS analysis; Figure S31. ESI-MS spectrum of **2** by UPLC-MS analysis; Figure S32. ESI-MS spectrum of **3** by UPLC-MS analysis; Figure S33. ESI-MS spectrum of **4** by UPLC-MS analysis; Figure S34. ESI-MS spectrum of **5** by UPLC-MS analysis; Figure S35. UV spectra of **1**, **2**, and **3** by UPLC-MS analysis; Figure S36. UV spectra of **4** and **5** by UPLC-MS analysis; Table S1. Physicochemical properties of **1**–**5**; Table S2. Comparison of NMR chemical shifts of **1** with reported compounds, solophenol A and nymphaeol C; Table S3. Comparison of NMR chemical shifts of **2** with reported compound, nymphaeol B; Table S4. Comparison of NMR chemical shifts of **3** with reported compound, schizolaenone C; Table S5. Comparison of NMR chemical shifts of **4** with reported compound, SB-202742; Table S6. Comparison of NMR chemical shifts of **5** with reported compound, 6-heptadeca-9Z,12Z-dienyl salicylic acid.

## Sample Availability

Samples of the compounds **1**−**5** are available from the authors.
