# Supplementary material for: In Vitro and In Silico Anti-Acetylcholinesterase Activity from Macaranga tanarius and Syzygium jambos
Source: Molecules. 2022 Apr 20;27(9):2648. doi: 10.3390/molecules27092648 (PMC9102781; doi:10.3390/molecules27092648)
Supplement: Supplementary file 1 [file molecules-27-02648-s001.zip › molecules-1640619-supplementary.pdf]

Supplementary information

In Vitro and In Silico Anti-Acetylcholinesterase Activity from *Macaranga tanarius* and *Syzygium jambos*

Mira Syahfriena Amir Rawa [1,2,3], Nurul Amira Nurul Azman [3], Suriani Mohamad [3], Toshihiko Nogawa [2,3,*] and Habibah A. Wahab [1,3,*]

1 Collaborative Laboratory for Herbal Standardization (CHEST), School of Pharmaceutical Sciences,
Universiti Sains Malaysia, 11800 Penang, Malaysia; mirasyah@usm.my

2 Chemical Biology Research Group, RIKEN Center for Sustainable Resource Science, 2-1 Hirosawa, Wako, Saitama, 351-0198, Japan

3 USM-RIKEN Interdisciplinary Collaboration for Advanced Sciences (URICAS), Universiti Sains Malaysia, 11800 Penang, Malaysia; nurulamira.nazman@gmail.com (N.A.N.A.); suriani@usm.my (S.M.)

* Correspondence: nogawat@riken.jp (T.N.); habibahw@usm.my (H.A.W.); Tel.: +81-048-467-9541 (T.N.);
+604-657-7888 (H.A.W.)

Contents:

Figure S1. Anti-acetylcholinesterase (AChE) activity of 18 potent Malaysian plant fractions (hexane, ethyl acetate, butanol, water) at 50 μg/mL final concentration. / page 4

Figure S2. Isolation scheme of *Macaranga tanarius* ethyl acetate extract. / page 5

Figure S3. Isolation scheme of *Syzygium jambos* hexane extract. / page 6

Figure S4. ^{1}H NMR spectrum of **1**. / page 7

Figure S5. ^{1}H NMR spectrum of **2**. / page 8

Figure S6. ^{1}H NMR spectrum of **3**. / page 9

Figure S7. ^{1}H NMR spectrum of **4**. / page 10

Figure S8. ^{1}H NMR spectrum of **5**. / page 11

Figure S9. ^{13}C NMR spectrum of **1.** / page 12

Figure S10. ^{13}C NMR spectrum of **2.** / page 13

Figure S11. ^{13}C NMR spectrum of **3.** / page 14

Figure S12. ^{13}C NMR spectrum of **4**. / page 15

Figure S13. ^{13}C NMR spectrum of **5.** / page 16

Figure S14. DEPT135 spectrum of **1**. / page 17

Figure S15. DEPT135 spectrum of **3**. / page 18

Figure S16. DEPT135 spectrum of **4**. / page 19

Figure S17. DEPT135 spectrum of **5**. / page 20

Figure S18. HSQC spectrum of **1**. / page 21

Figure S19. HSQC spectrum of **2**. / page 22

Figure S20. HSQC spectrum of **3**. / page 23

Figure S21. HSQC spectrum of **4**. / page 24

Figure S22. HSQC spectrum of **5**. / page 25

Figure S23. DQF-COSY spectrum of **1**. / page 26

Figure S24. DQF-COSY spectrum of **3**. / page 27

Figure S25. DQF-COSY spectrum of **5**. / page 28

Figure S26. HMBC spectrum of **1**. / page 29

Figure S27. HMBC spectrum of **3**. / page 30

Figure S28. HMBC spectrum of **4**. / page 31

Figure S29. HMBC spectrum of **5**. / page 32

Figure S30. ESI-MS spectrum of **1** by UPLC-MS analysis. / page 33

Figure S31. ESI-MS spectrum of **2** by UPLC-MS analysis. / page 34

Figure S32. ESI-MS spectrum of **3** by UPLC-MS analysis. / page 35

Figure S33. ESI-MS spectrum of **4** by UPLC-MS analysis. / page 36

Figure S34. ESI-MS spectrum of **5** by UPLC-MS analysis. / page 37

Figure S35. UV spectra of **1**, **2**, and **3** by UPLC-MS analysis. / page 38

Figure S36. UV spectra of **4** and **5** by UPLC-MS analysis. / page 39

Table S1. Physicochemical properties of **1** – **5**. / page 40

Table S2. Comparison of NMR chemical shifts of **1** with reported compounds, solophenol A and nymphaeol C. / page 41

Table S3. Comparison of NMR chemical shifts of **2** with reported compound, nymphaeol B. / page 42

Table S4. Comparison of NMR chemical shifts of **3** with reported compound, schizolaenone C. / page 43

Table S5. Comparison of NMR chemical shifts of **4** with reported compound, SB-202742. / page 44

Table S6. Comparison of NMR chemical shifts of **5** with reported compound, 6-heptadeca-9Z,12Z-dienyl salicylic acid./ page 45

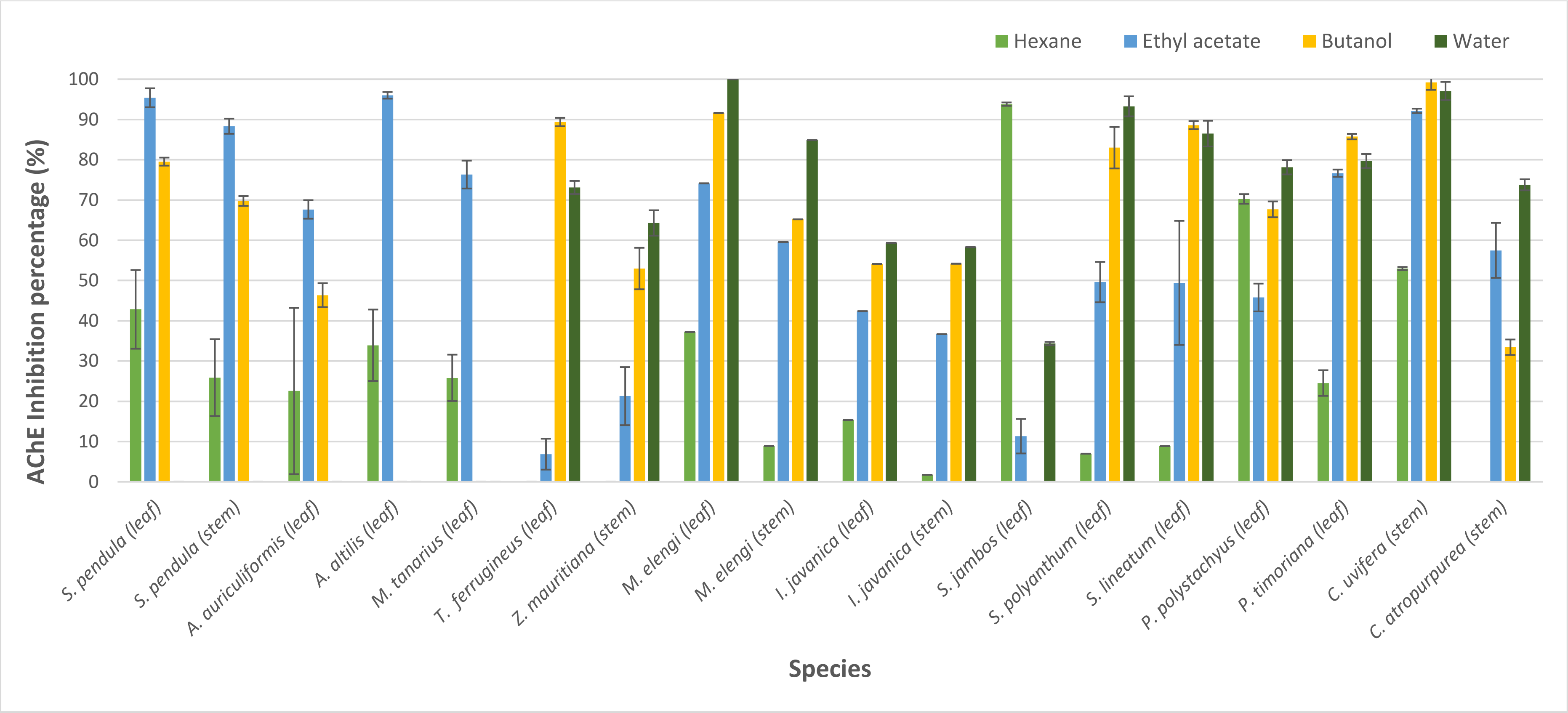

Figure S1. Anti-acetylcholinesterase (AChE) activity of 18 potent Malaysian plant fractions (hexane, ethyl acetate, butanol, water) at 50 µg/mL final concentration [10]. *M. tanarius* and *S. jambos* were chosen for isolation based on their low molecular weight active constituents with no report of identification of AChE inhibitors from these species before.

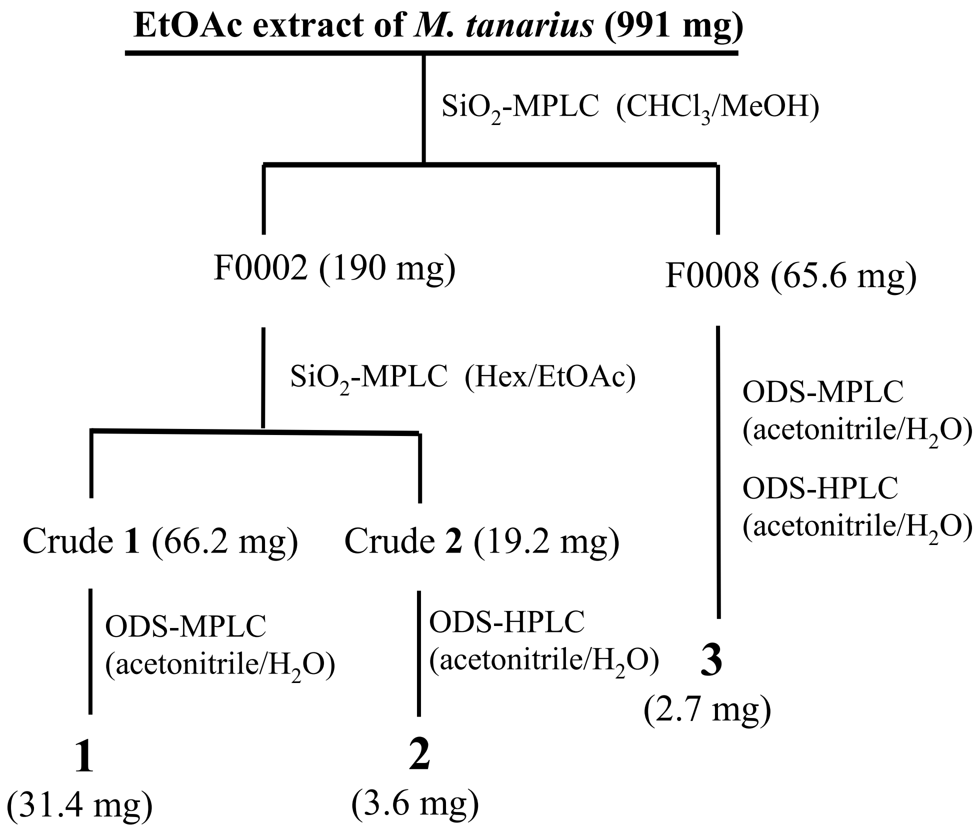

Figure S2. Isolation scheme of *Macaranga tanarius* ethyl acetate extract.

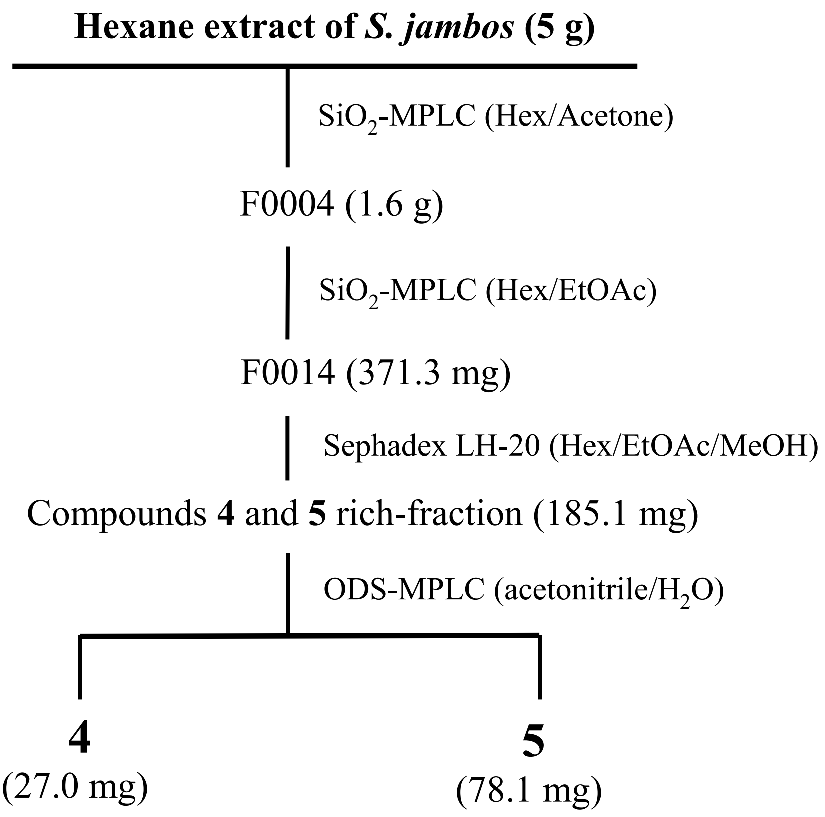

Figure S3. Isolation scheme of *Syzygium jambos* hexane extract.

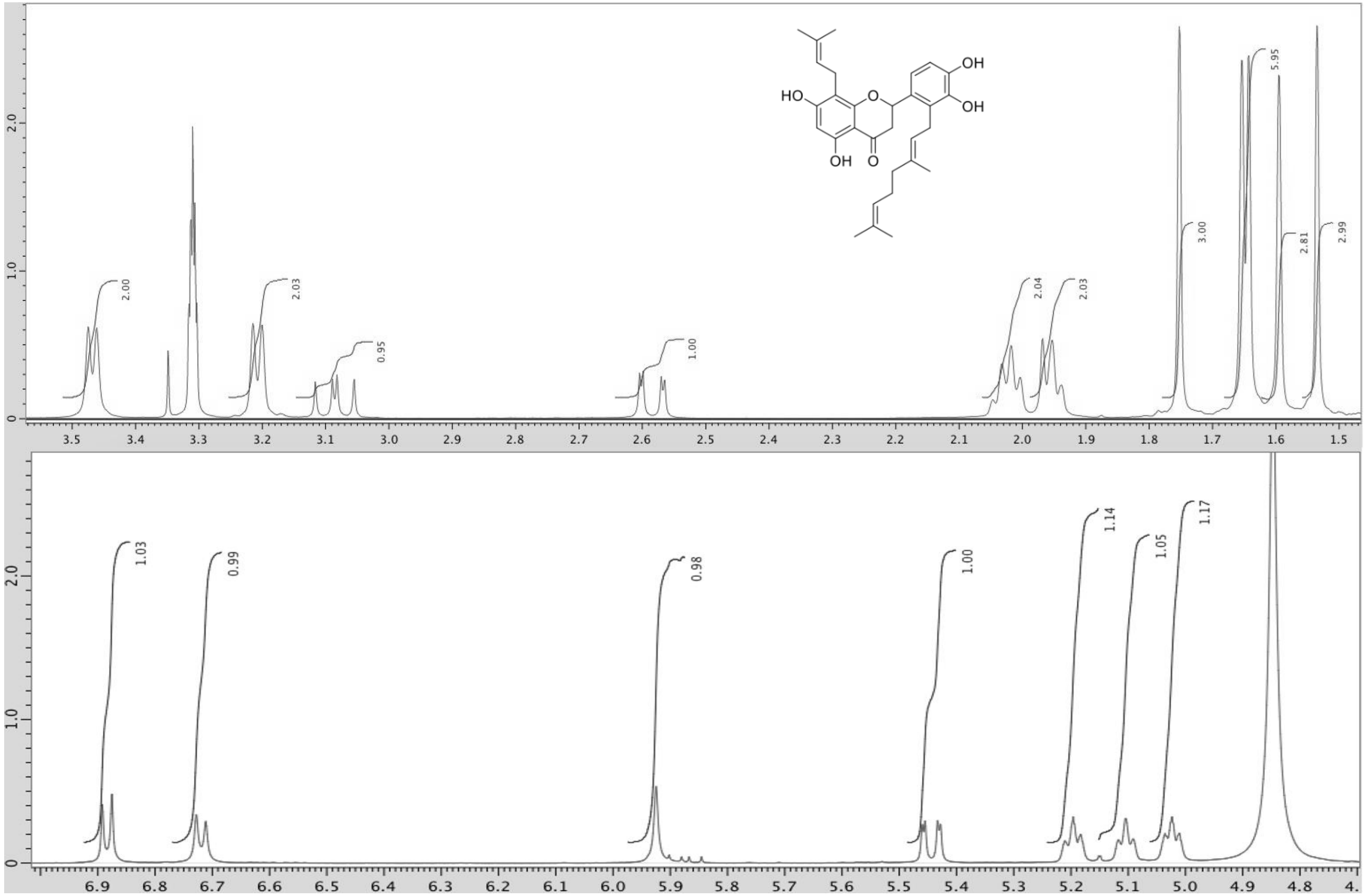

Figure S4. ^{1}H-NMR spectrum of **1.**

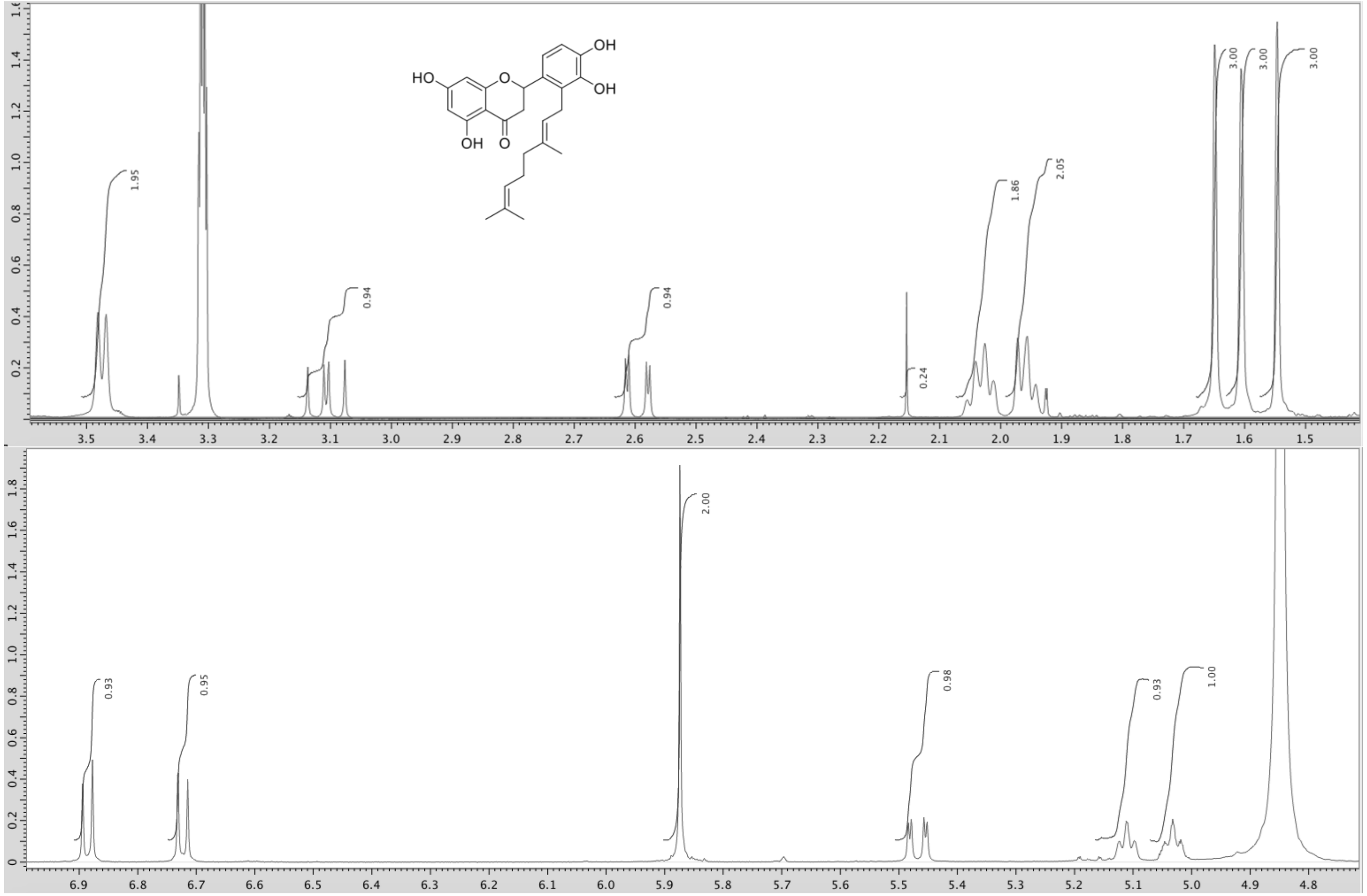

Figure S5. ¹H-NMR spectrum of **2.**

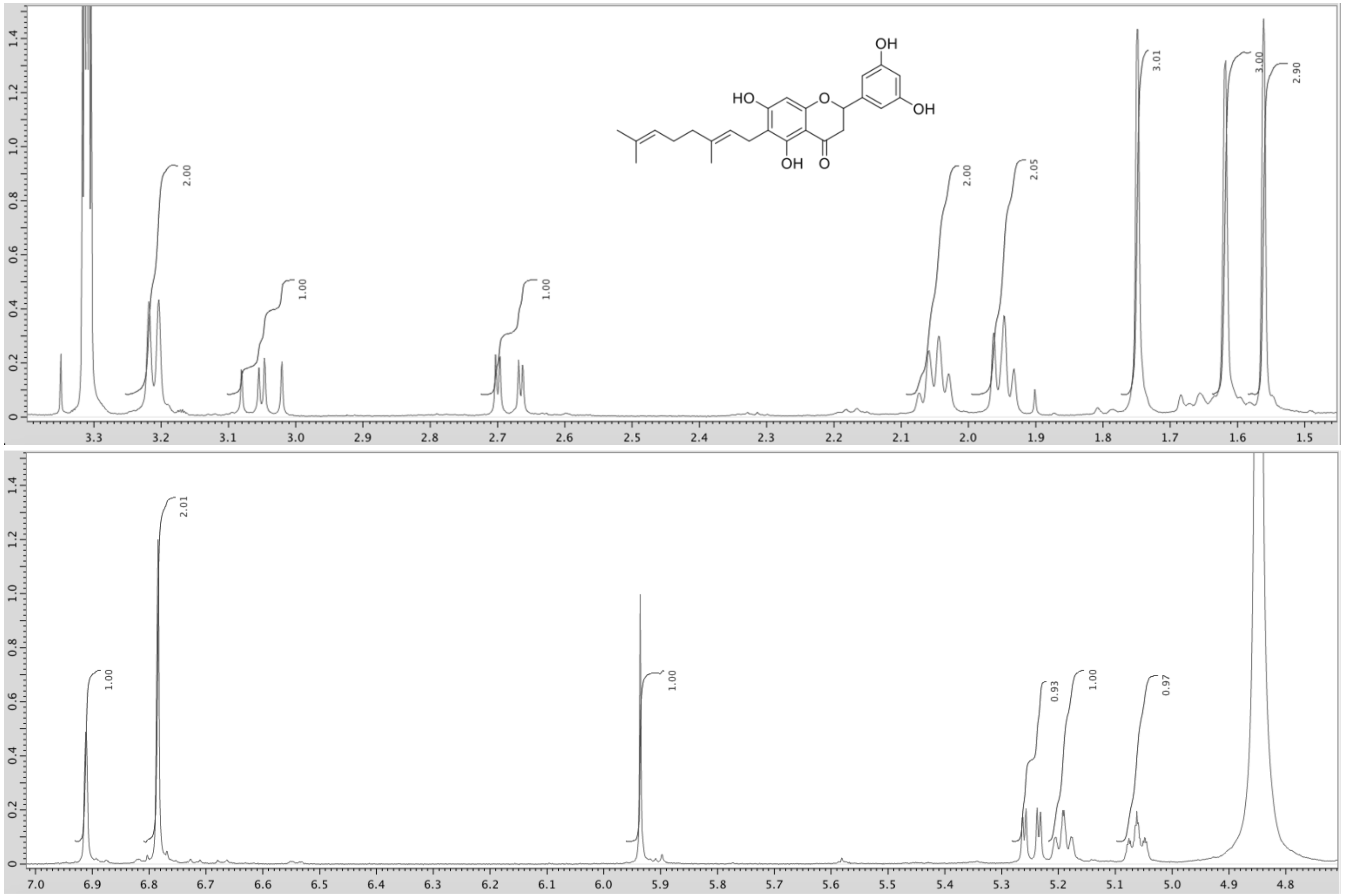

Figure S6. ^{1}H-NMR spectrum of **3.**

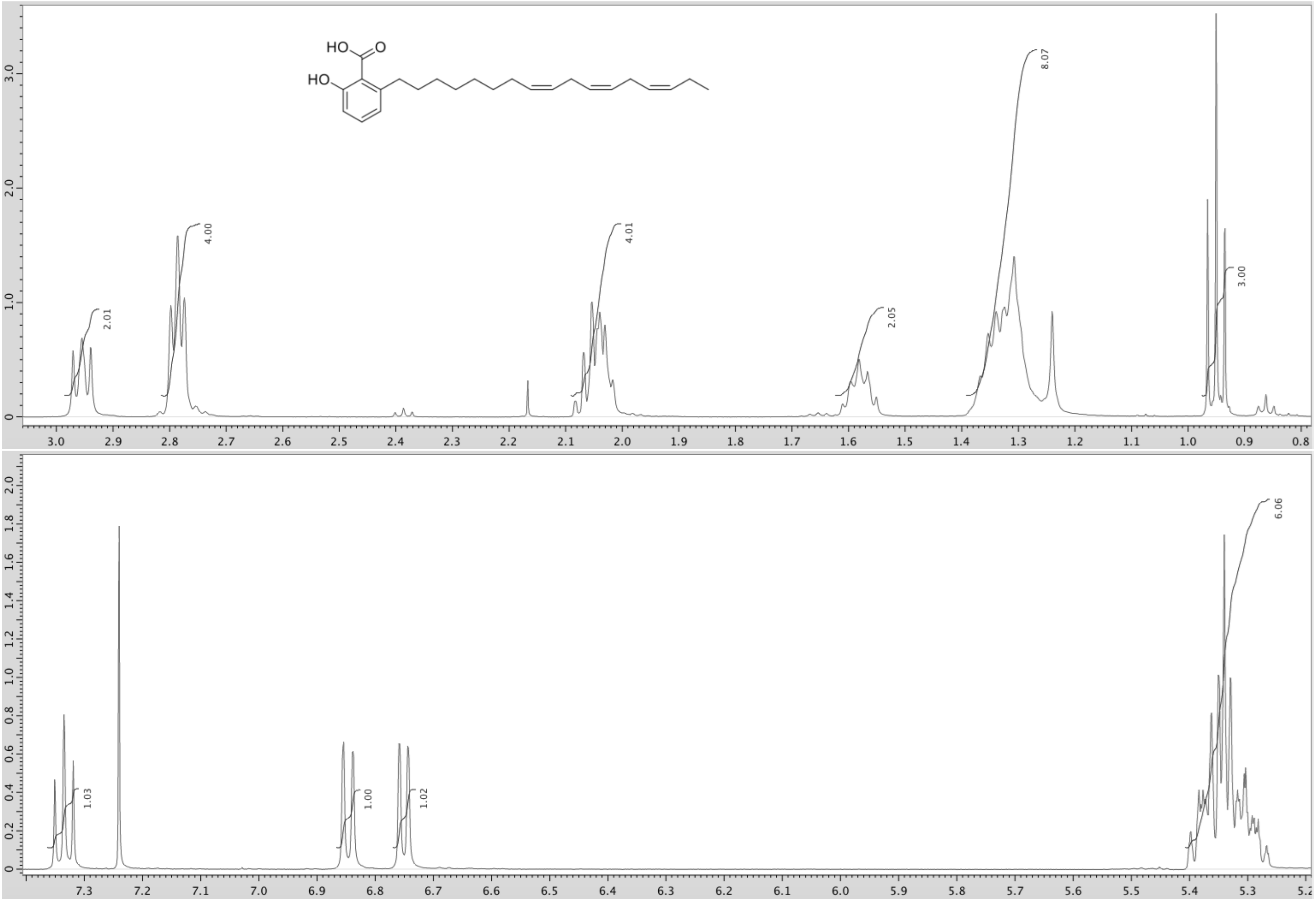

Figure S7. ^{1}H-NMR spectrum of **4.**

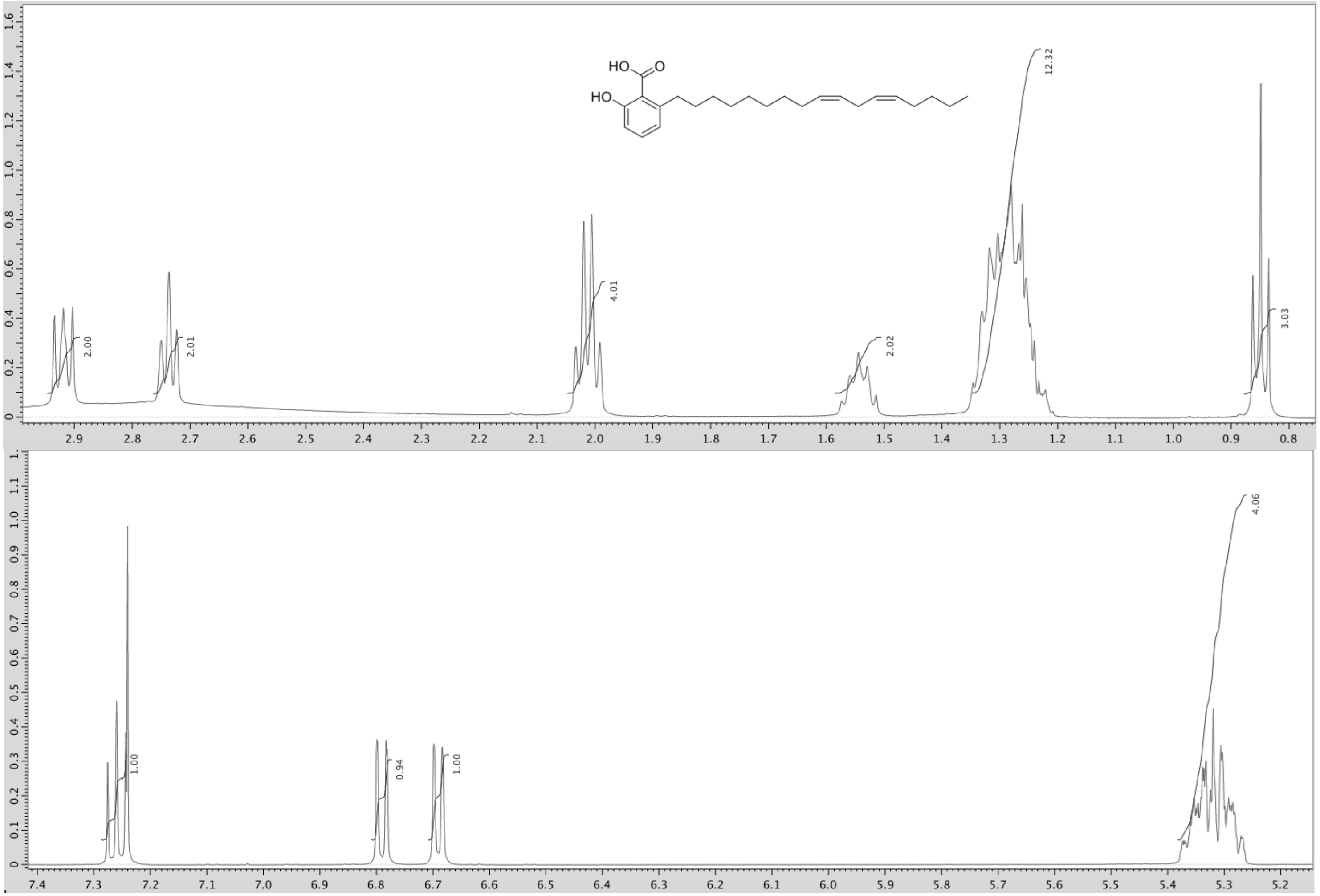

Figure S8. ^{1}H-NMR spectrum of **5.**

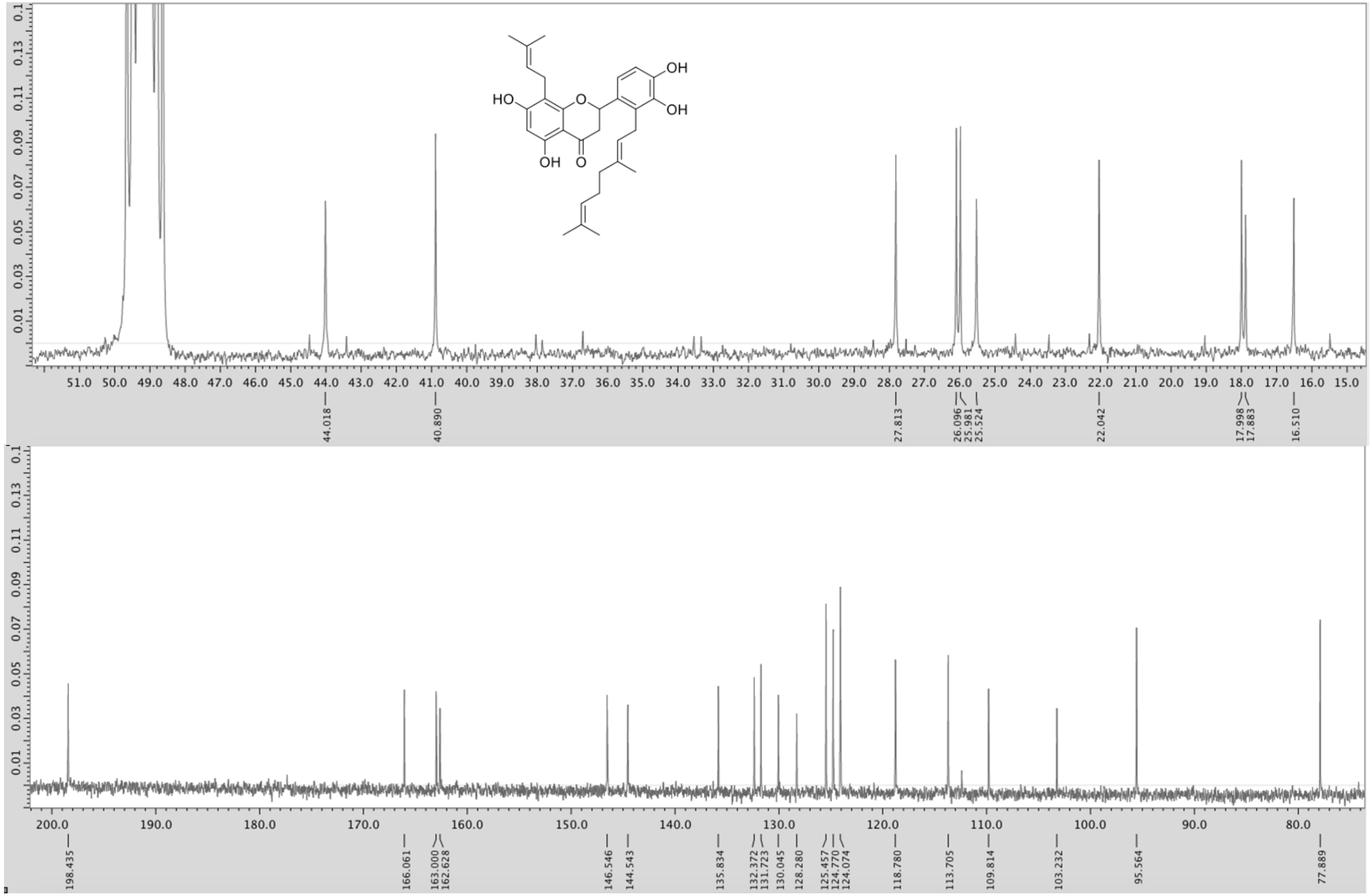

Figure S9. ^{13}C-NMR spectrum of **1.**

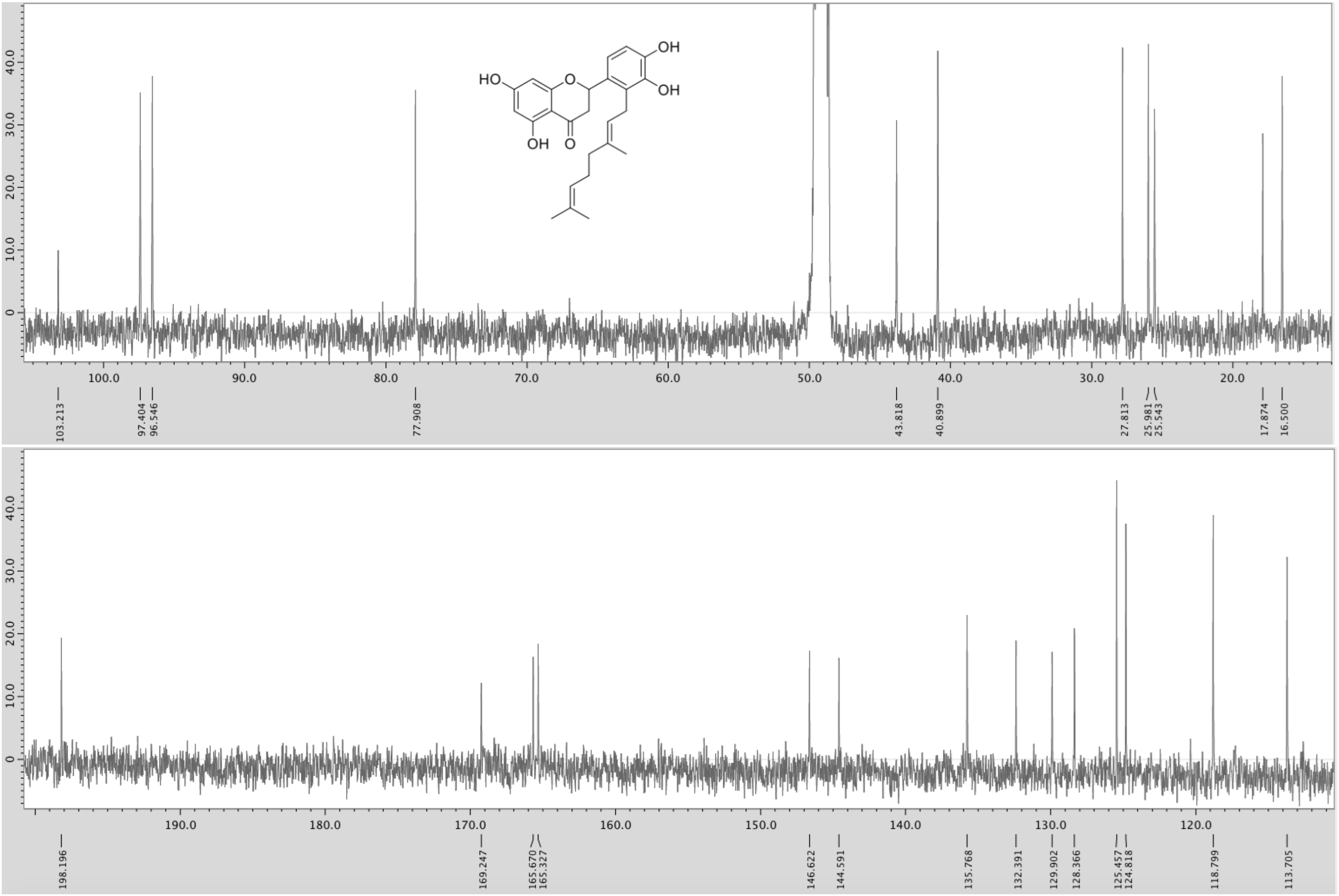

Figure S10. ^{13}C-NMR spectrum of **2.**

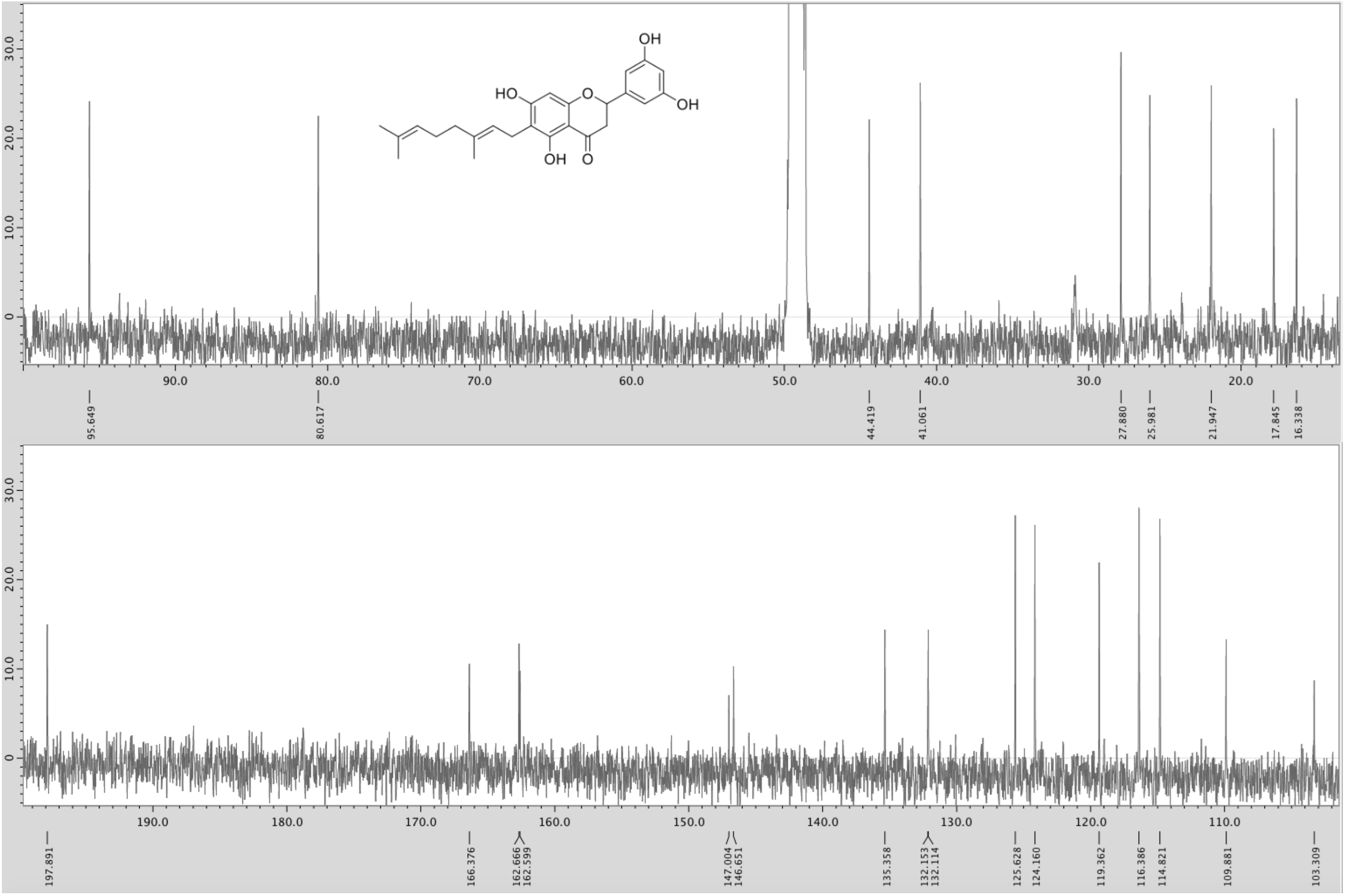

Figure S11. ^{13}C-NMR spectrum of **3.**

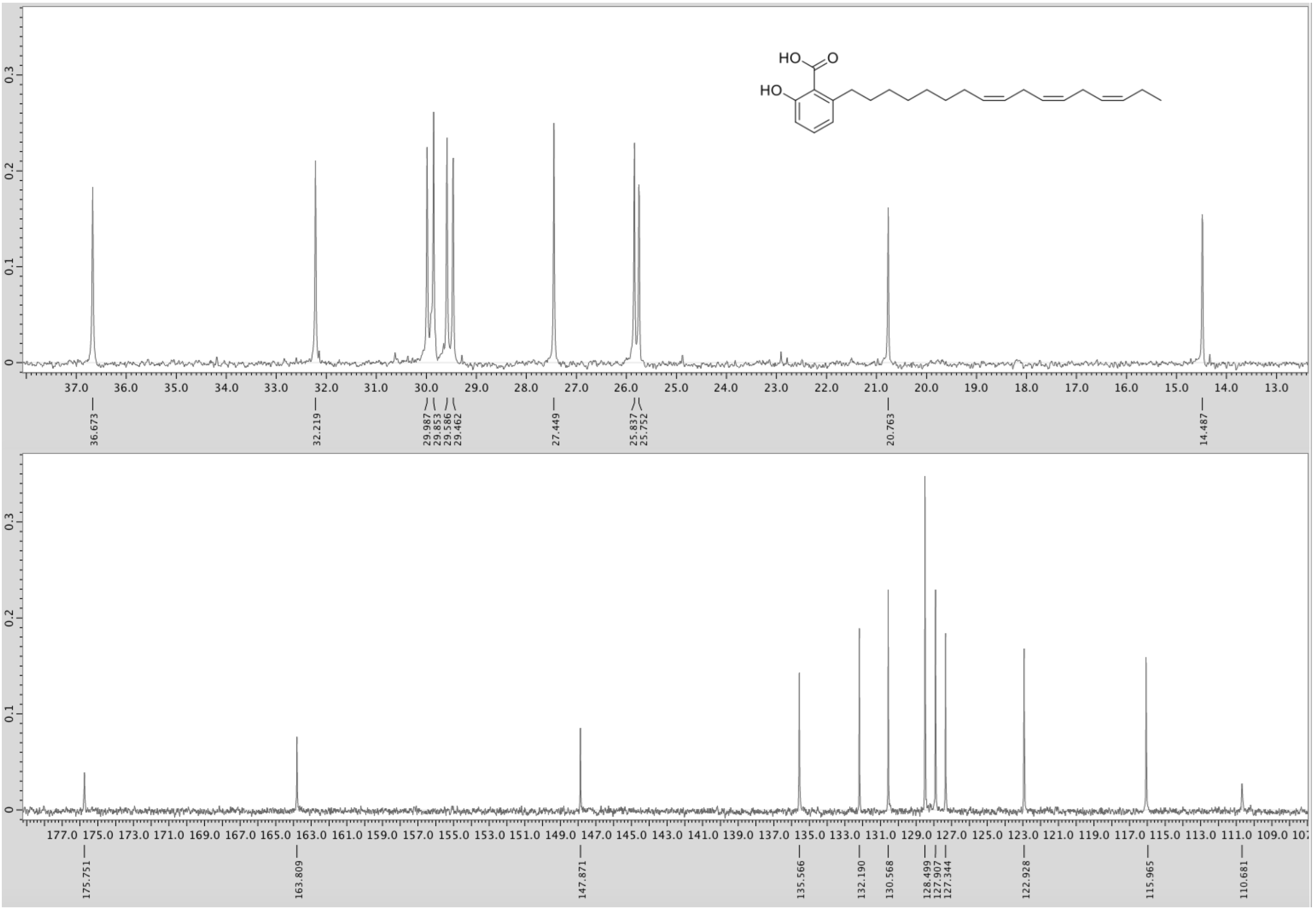

Figure S12. ^{13}C-NMR spectrum of **4.**

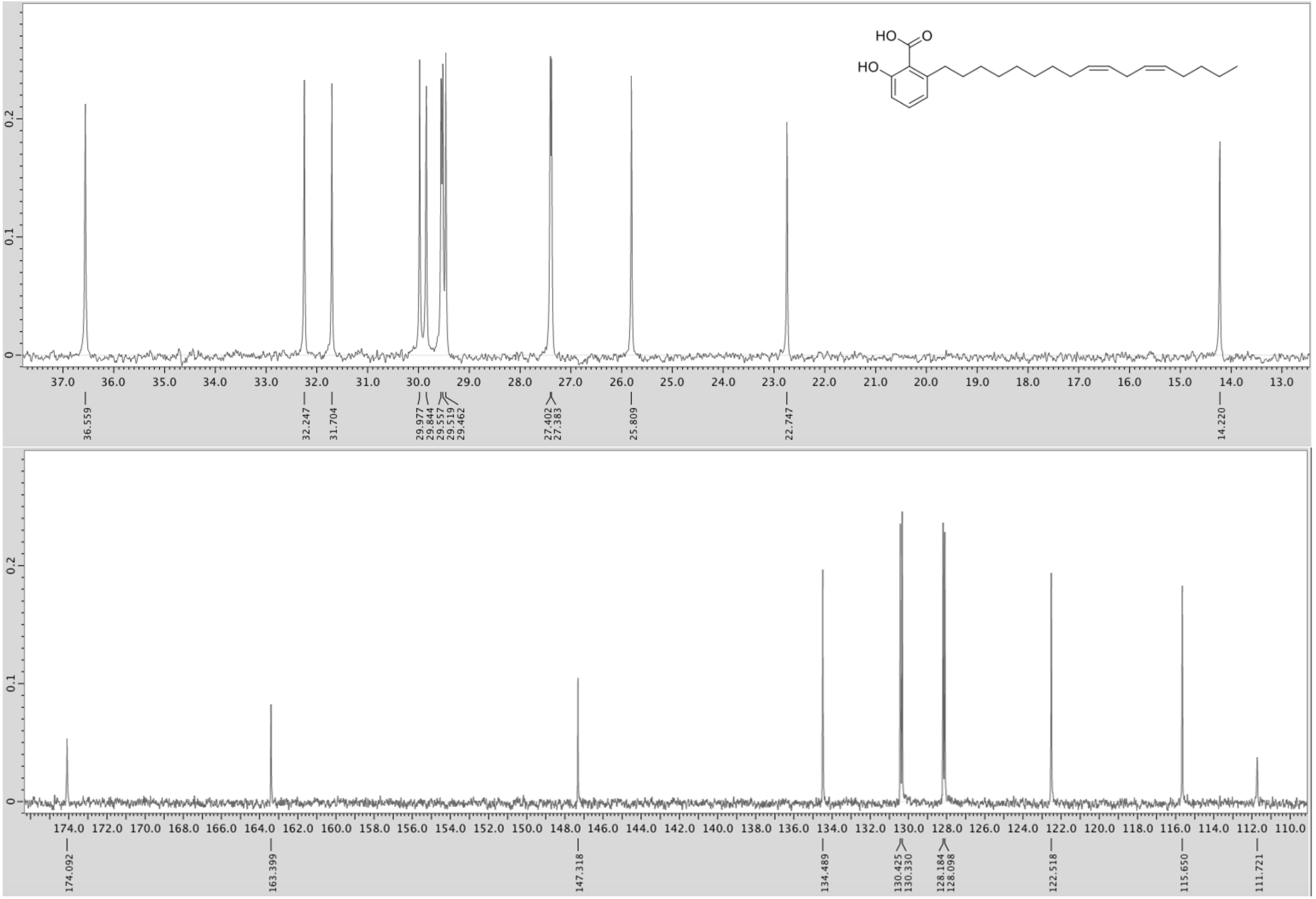

Figure S13. ^{13}C-NMR spectrum of **5.**

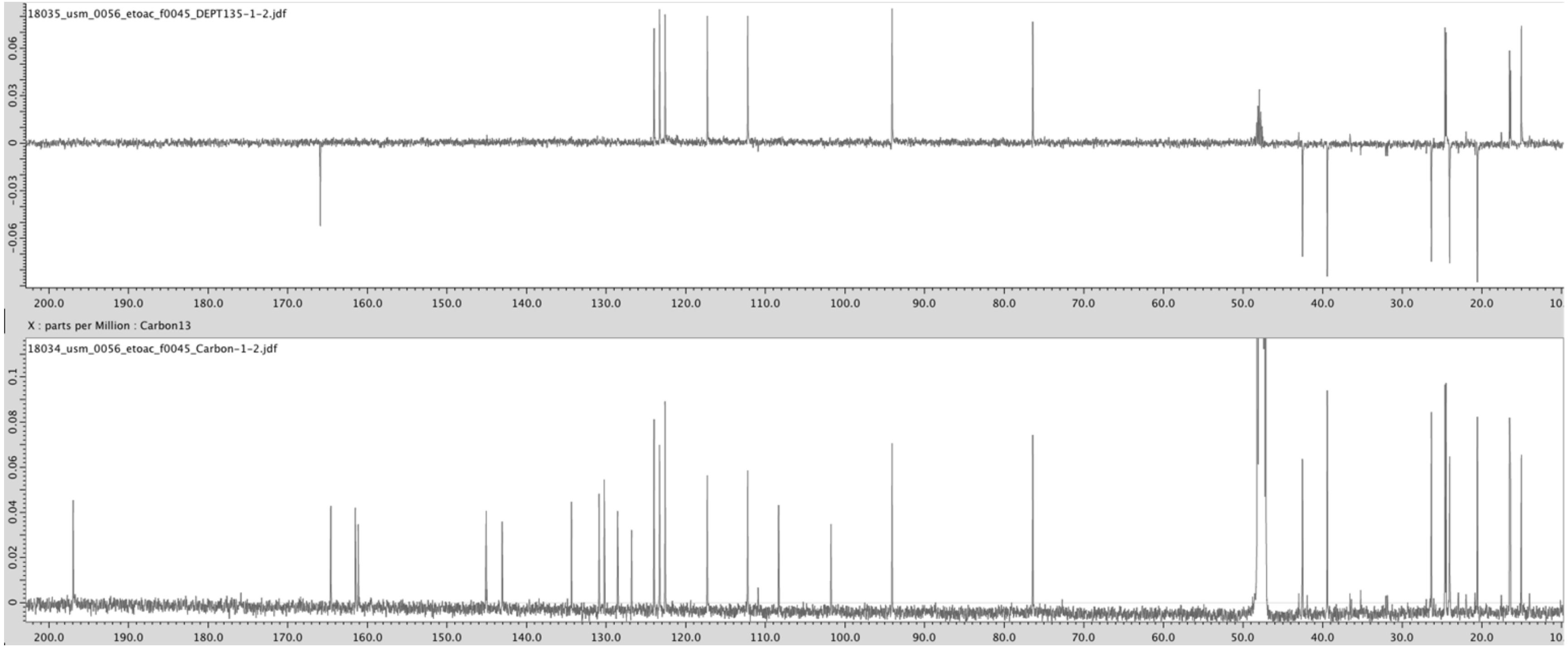

Figure S14. DEPT-135 spectrum of **1.**

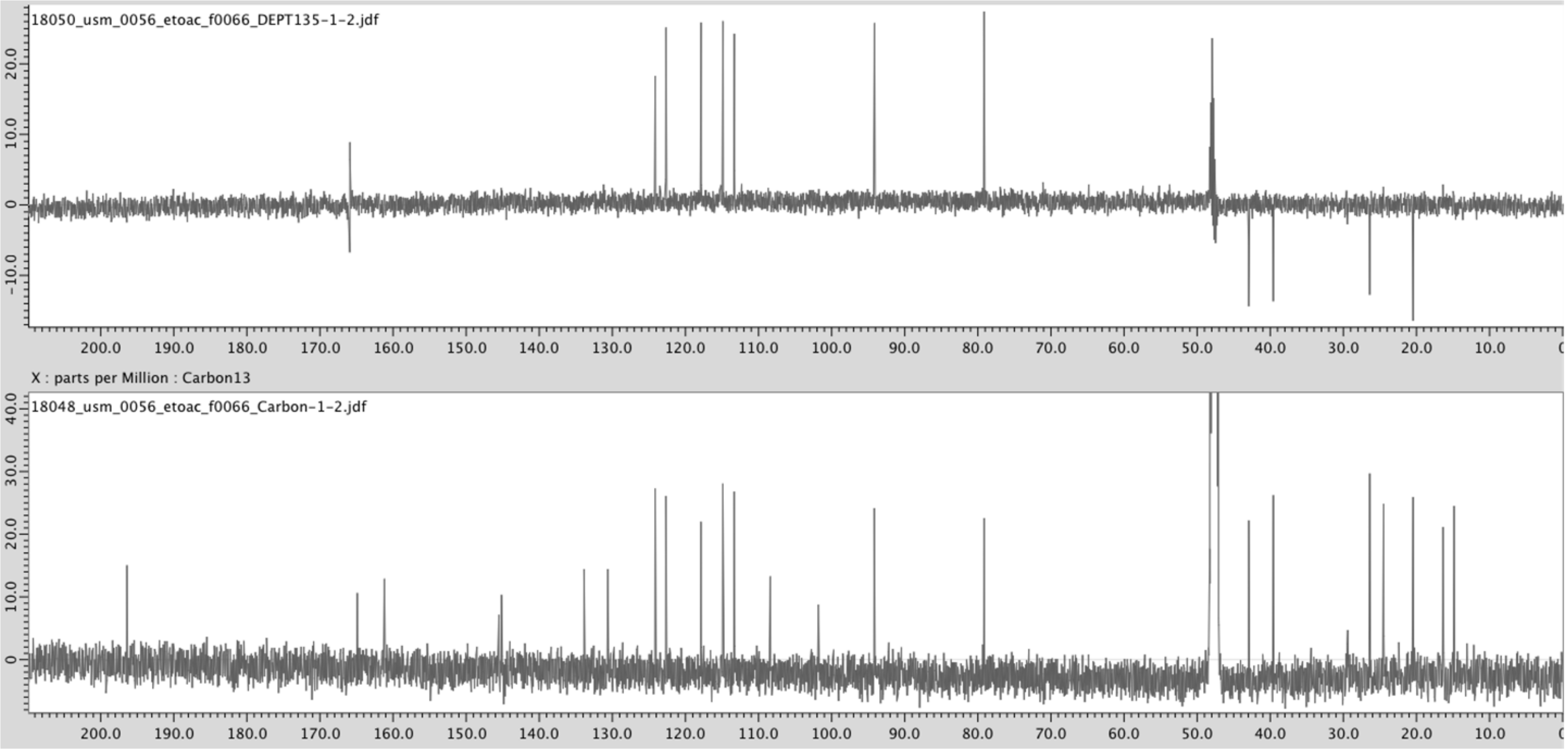

Figure S15. DEPT-135 spectrum of **3.**

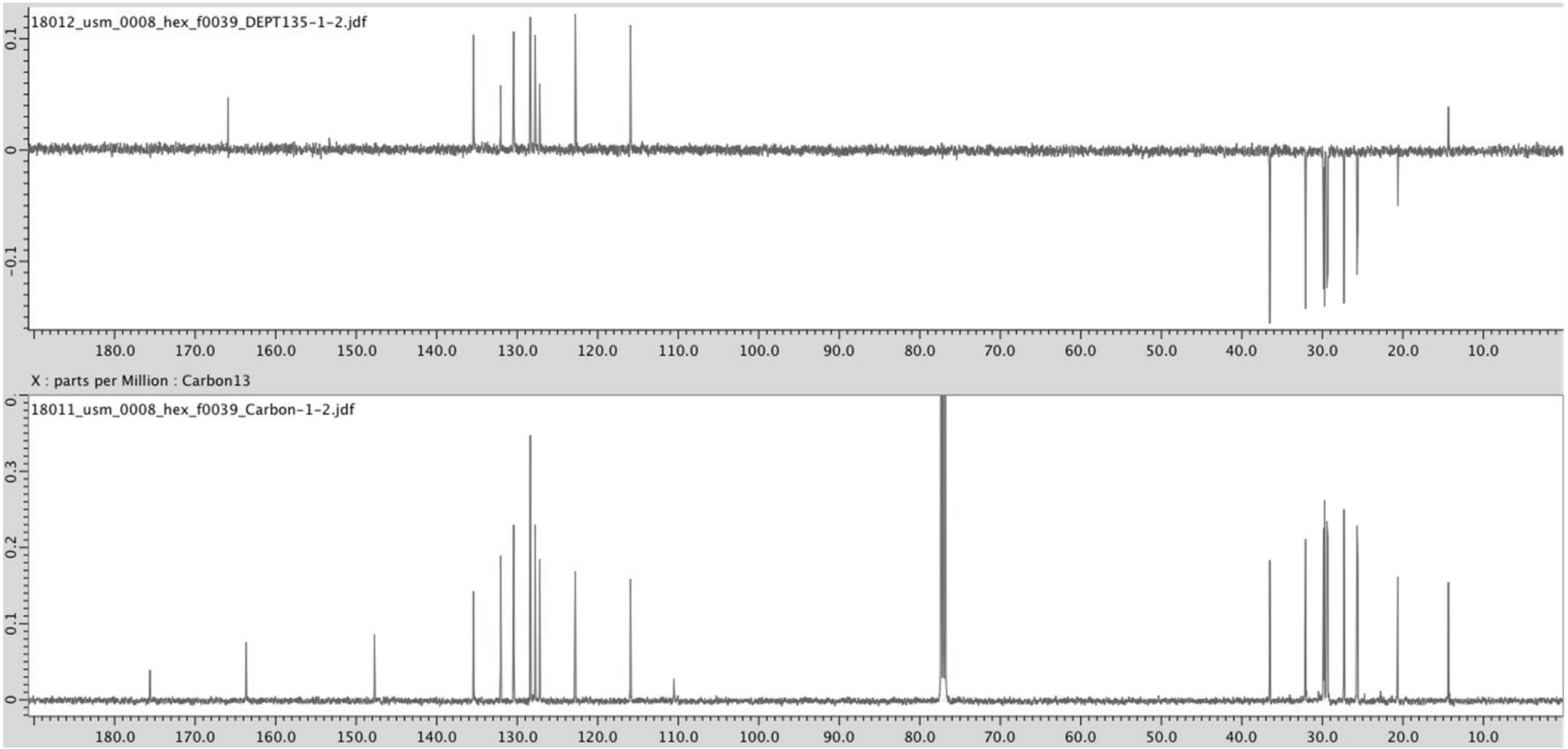

Figure S16. DEPT-135 spectrum of **4.**

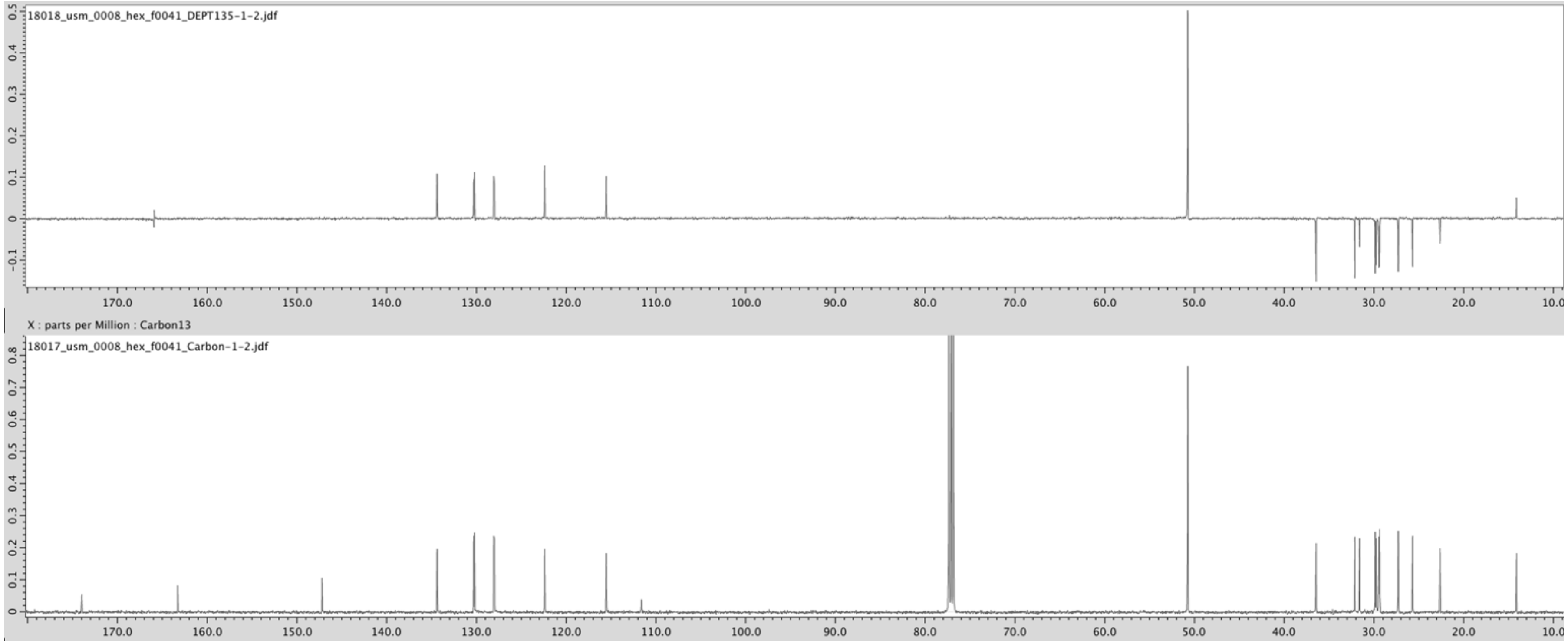

Figure S17. DEPT-135 spectrum of **5.**

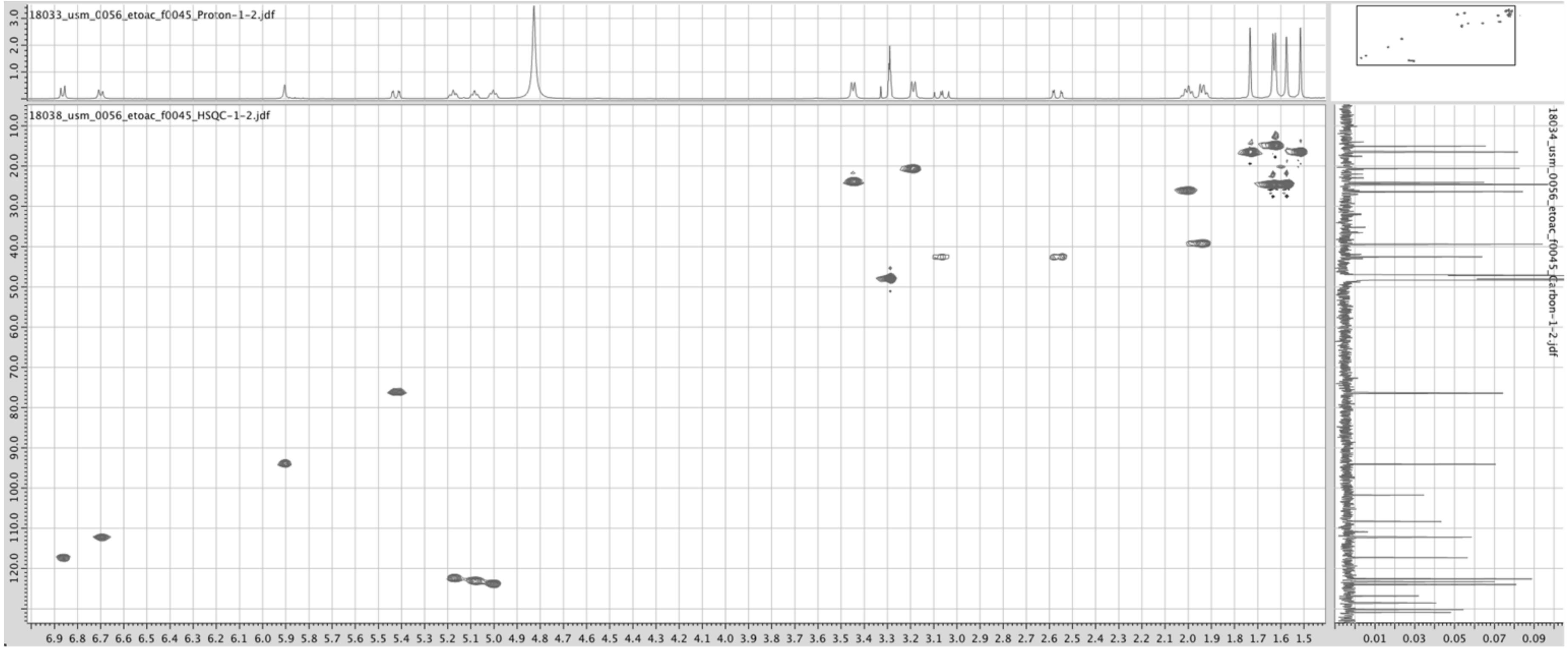

Figure S18. HSQC spectrum of **1.**

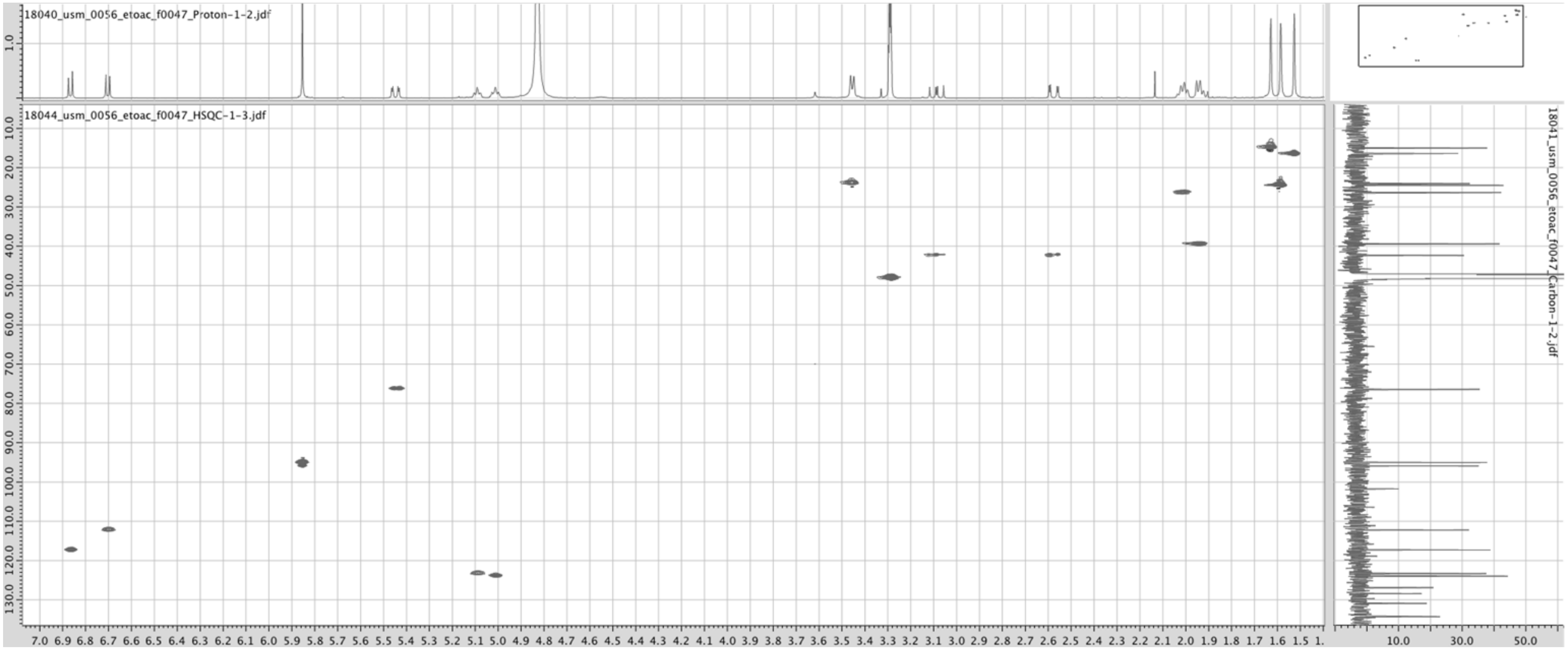

Figure S19. HSQC spectrum of **2.**

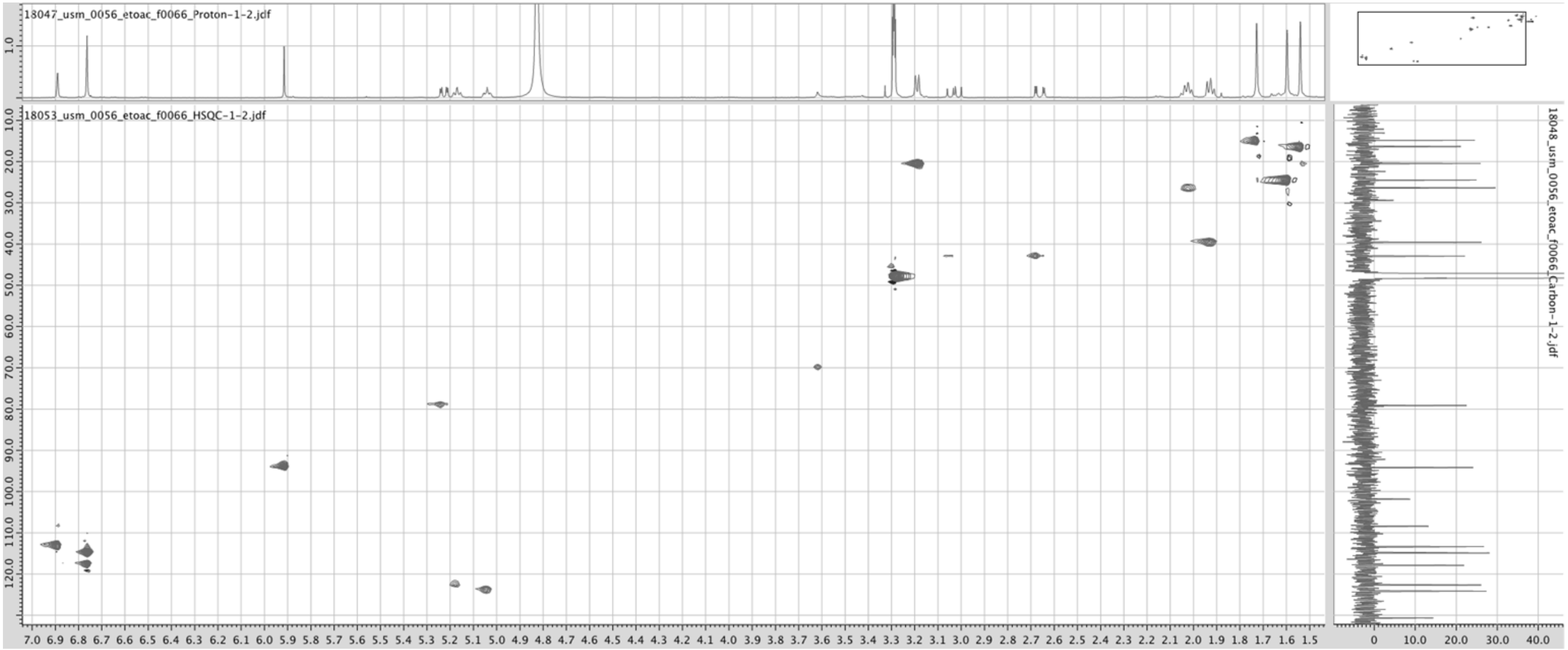

Figure S20. HSQC spectrum of **3.**

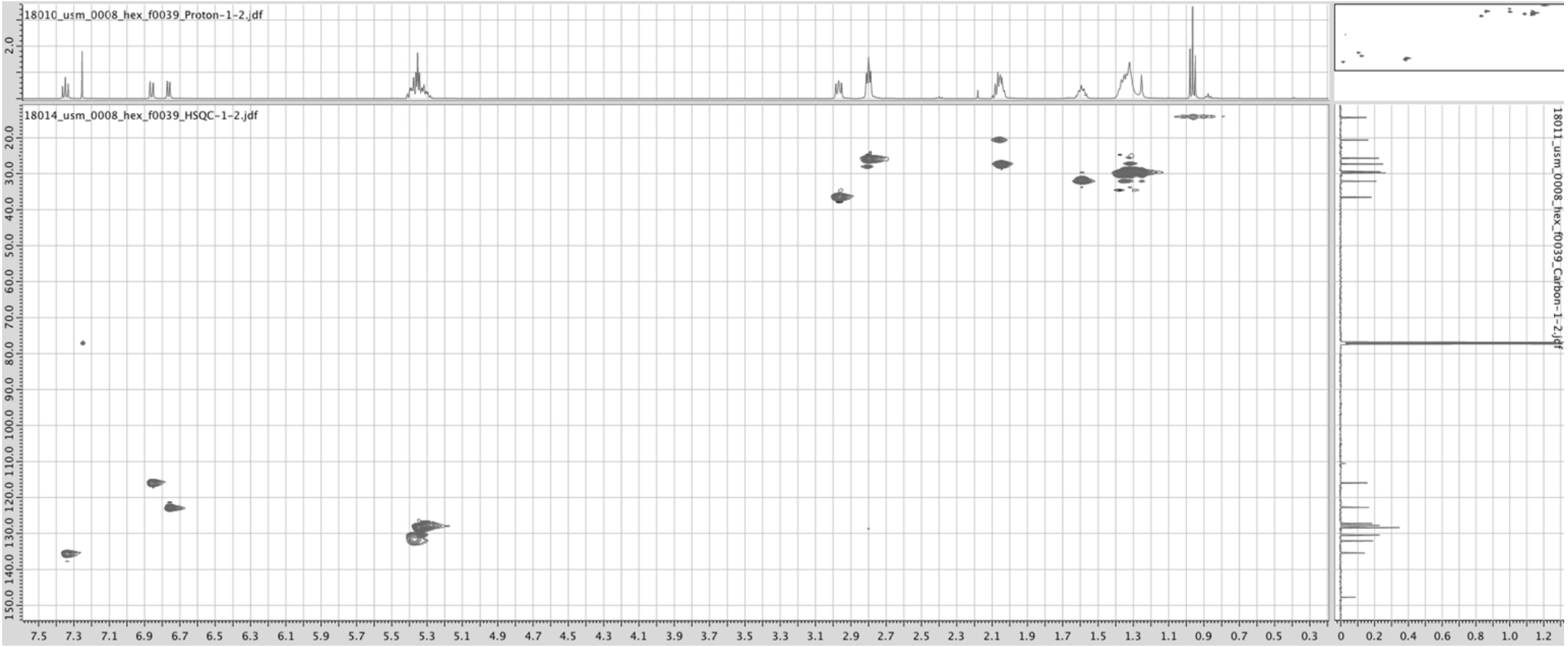

Figure S21. HSQC spectrum of **4.**

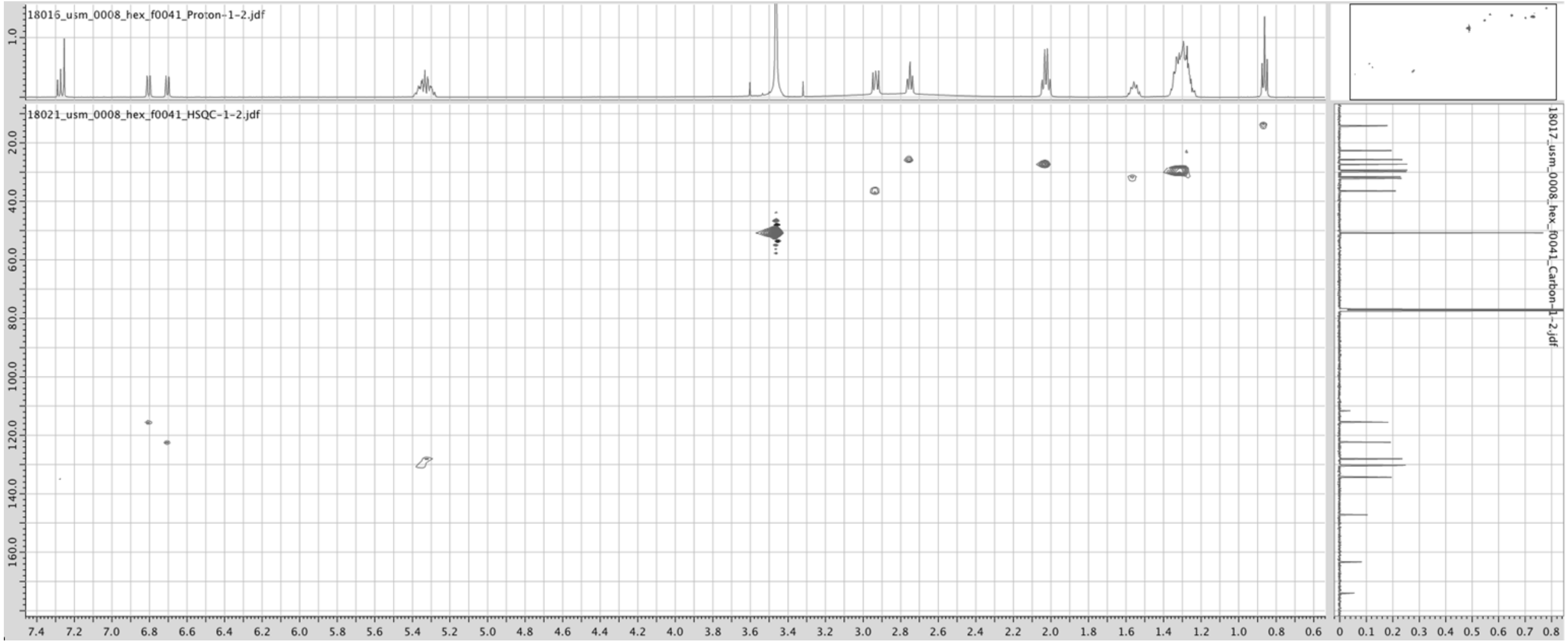

Figure S22. HSQC spectrum of **5.**

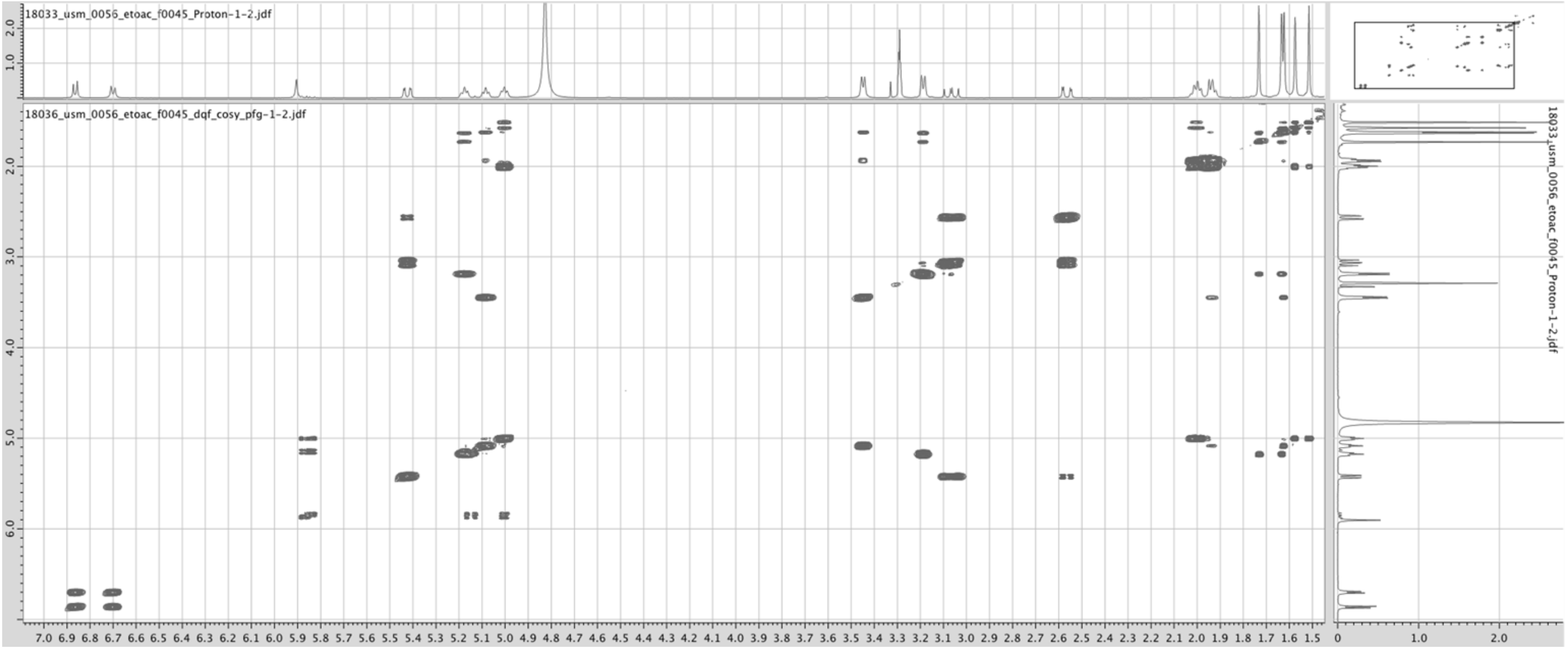

Figure S23. DQF-COSY spectrum of **1.**

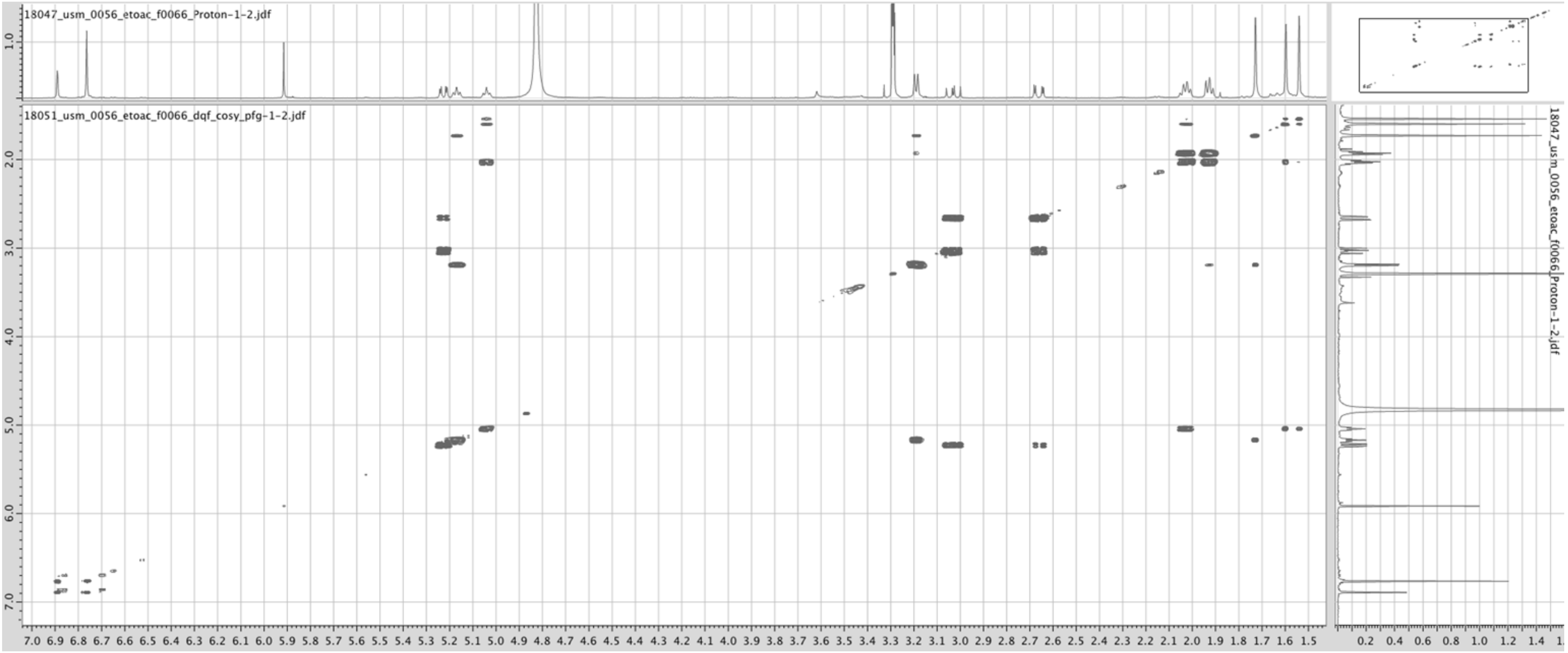

Figure S24. DQF-COSY spectrum of **3.**

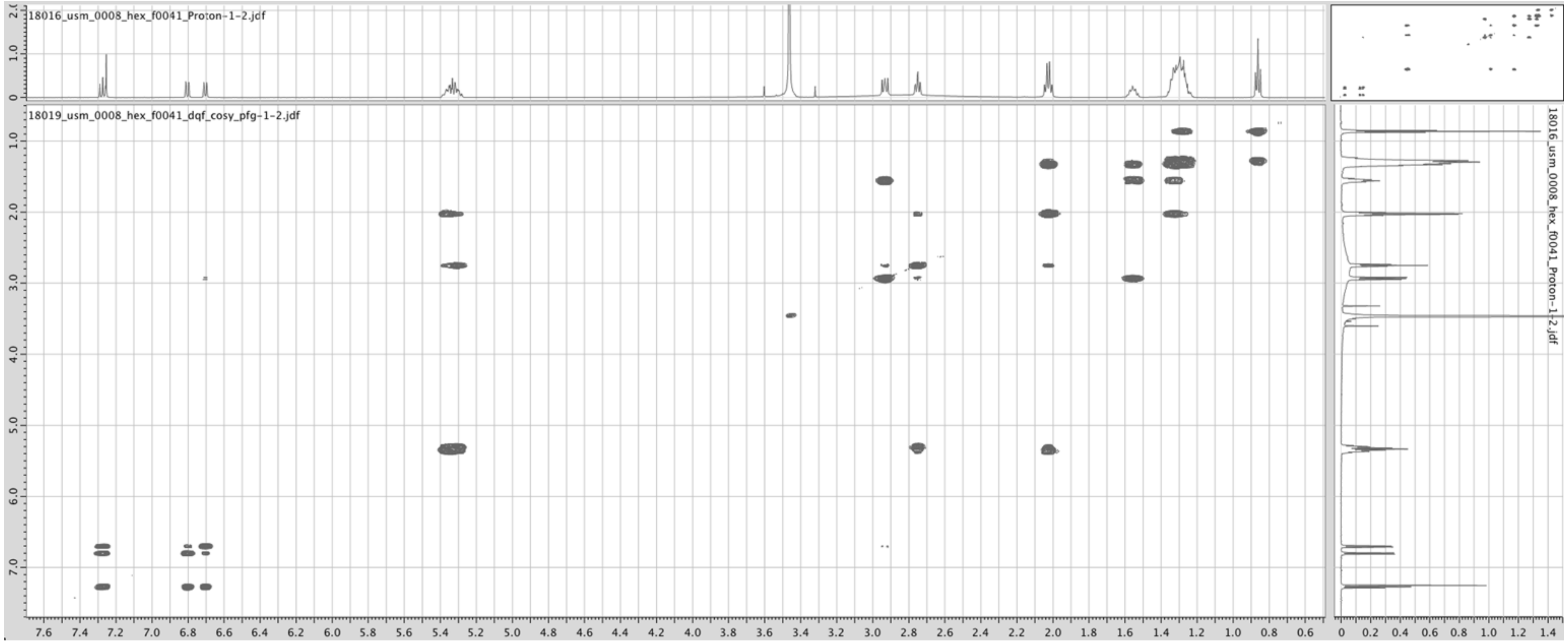

Figure S25. DQF-COSY spectrum of **5.**

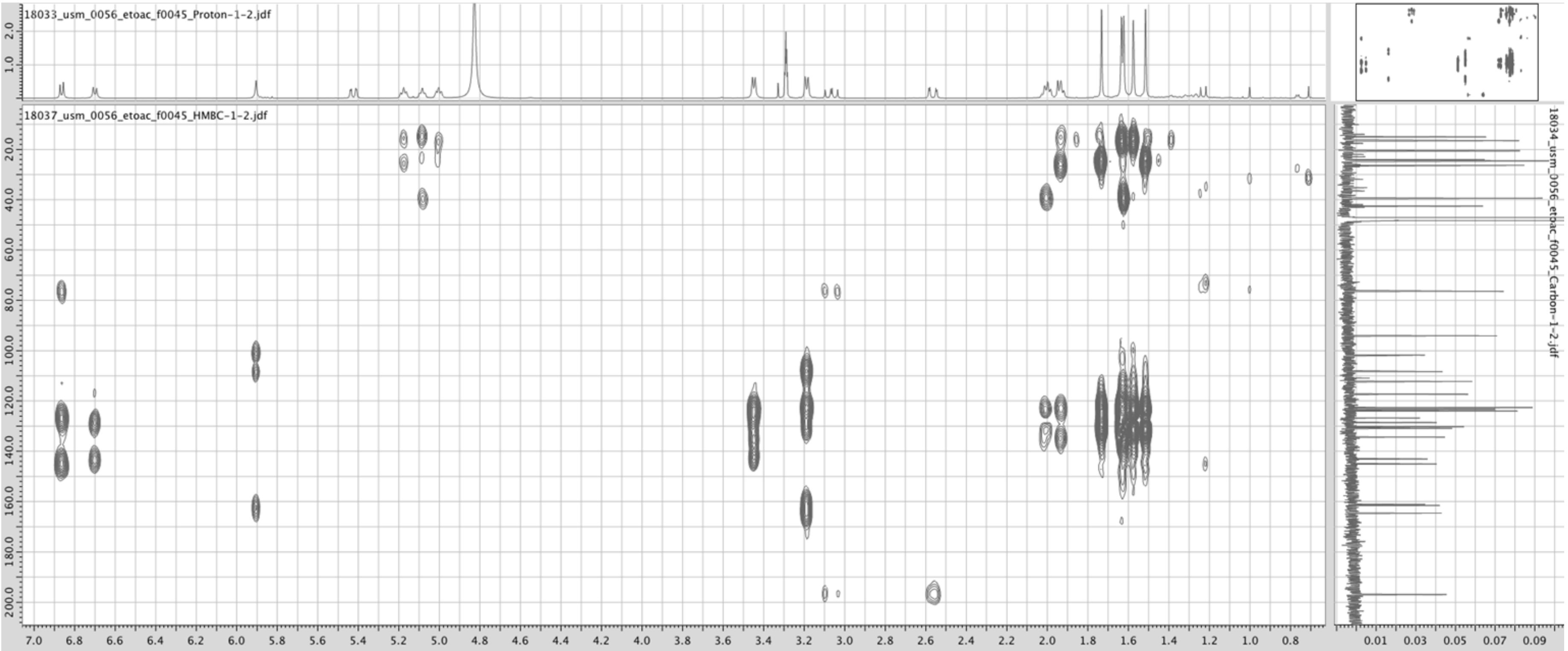

Figure S26. HMBC spectrum of **1.**

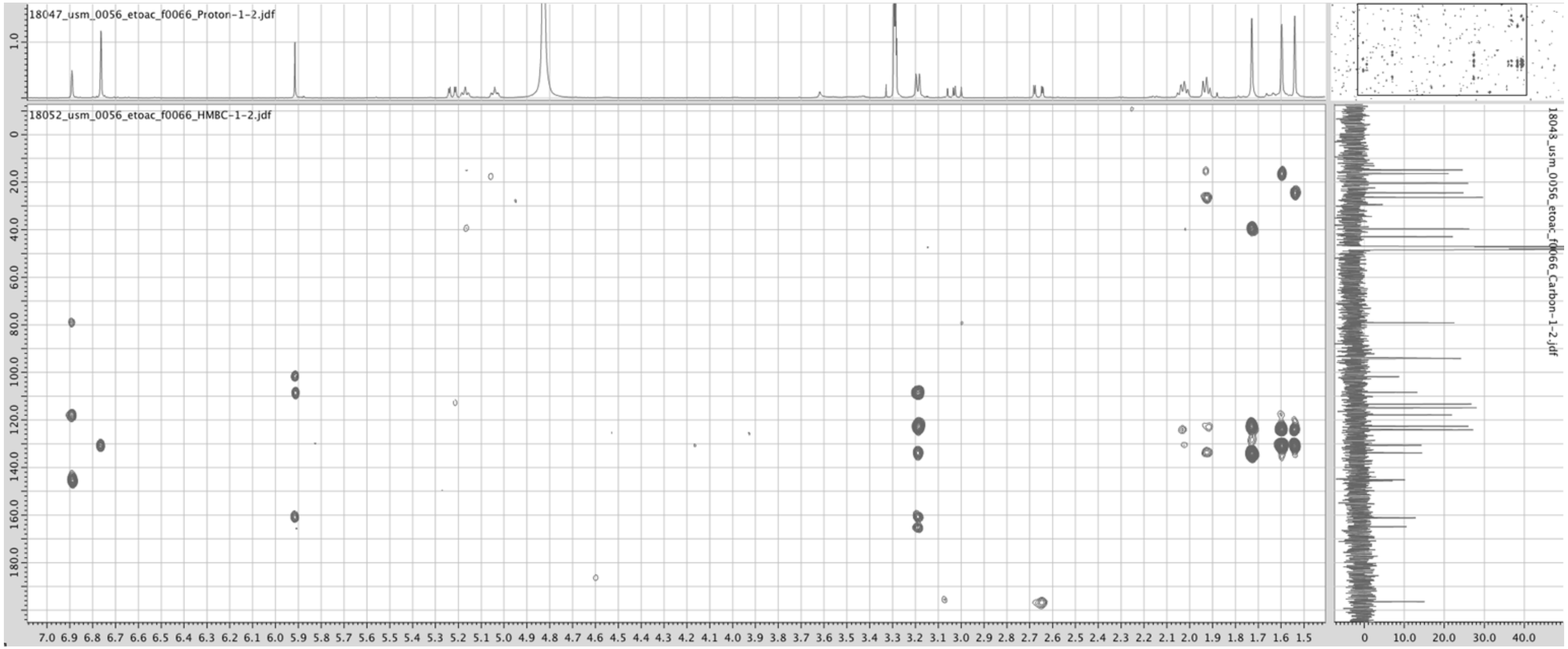

Figure S27. HMBC spectrum of **3.**

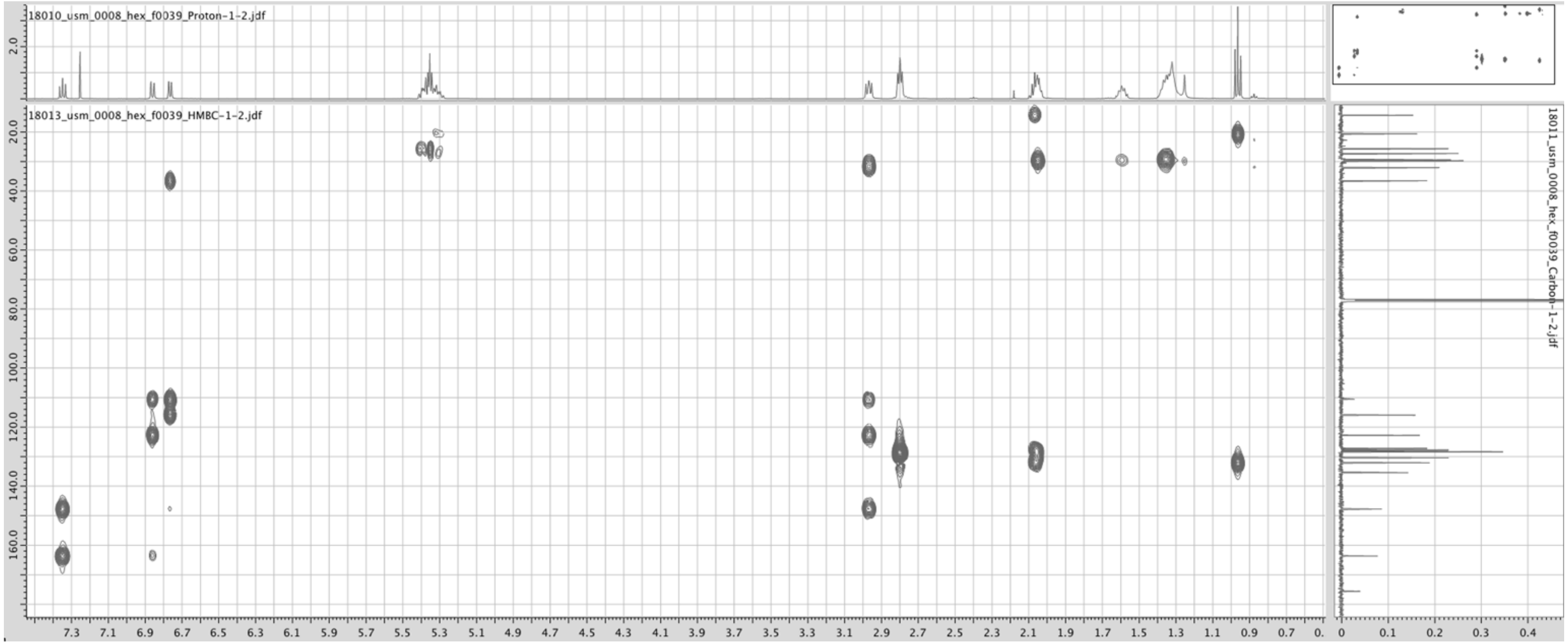

Figure S28. HMBC spectrum of **4.**

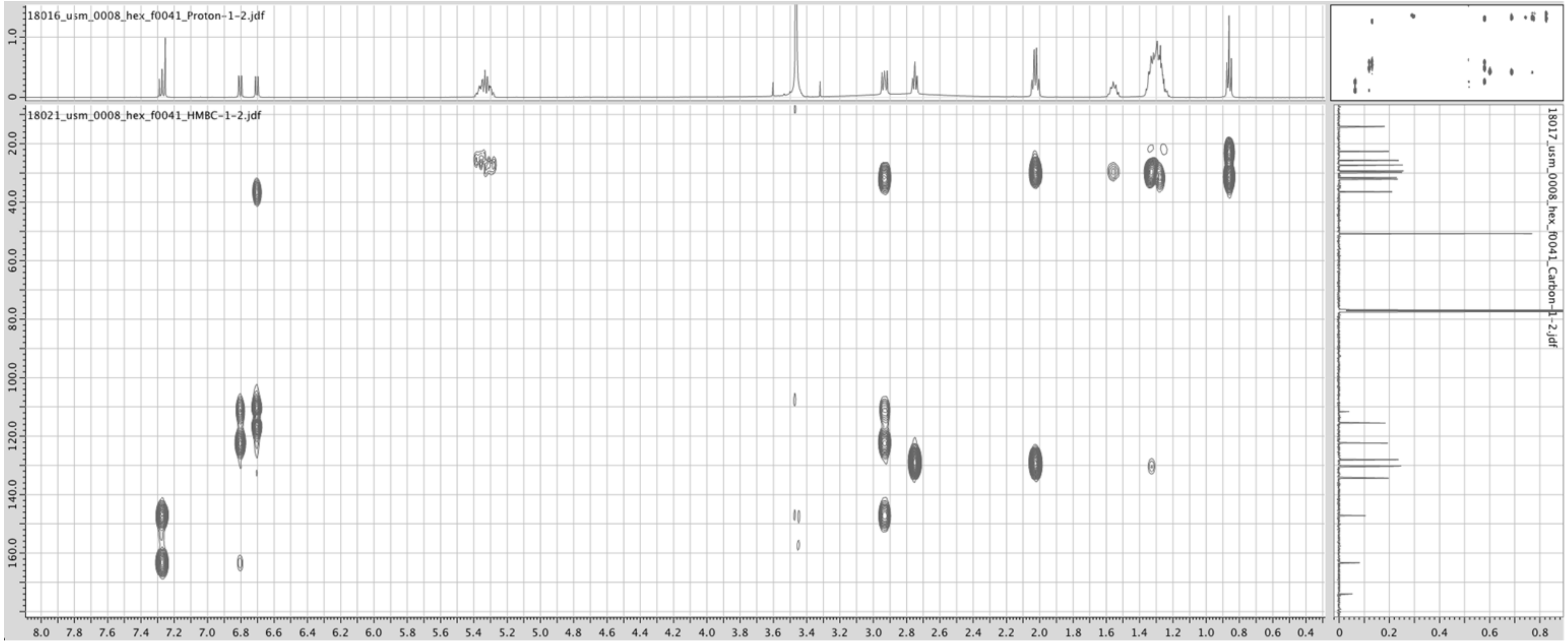

Figure S29. HMBC spectrum of **5.**

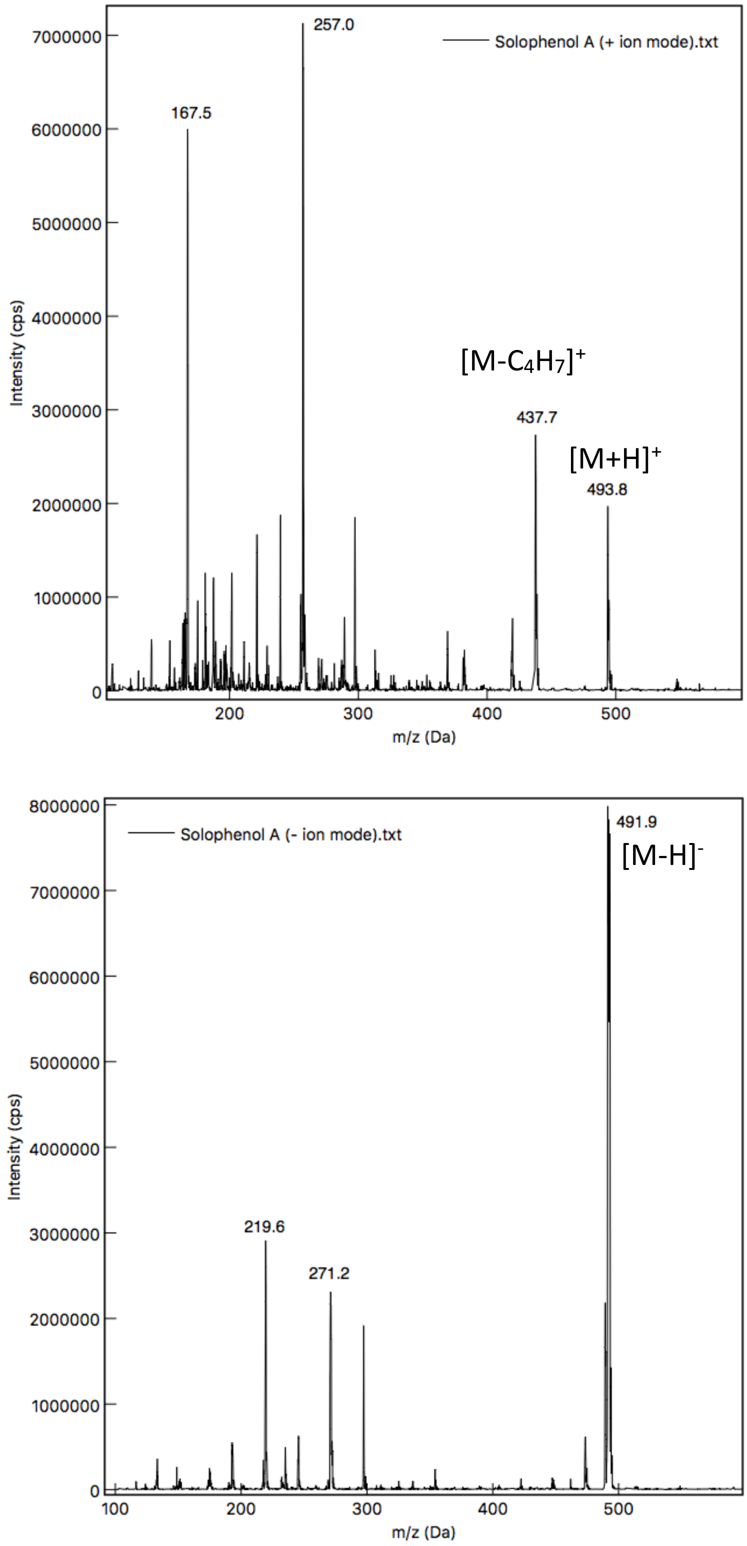

Figure S30. ESI-MS spectrum of **1** by UPLC-MS analysis.

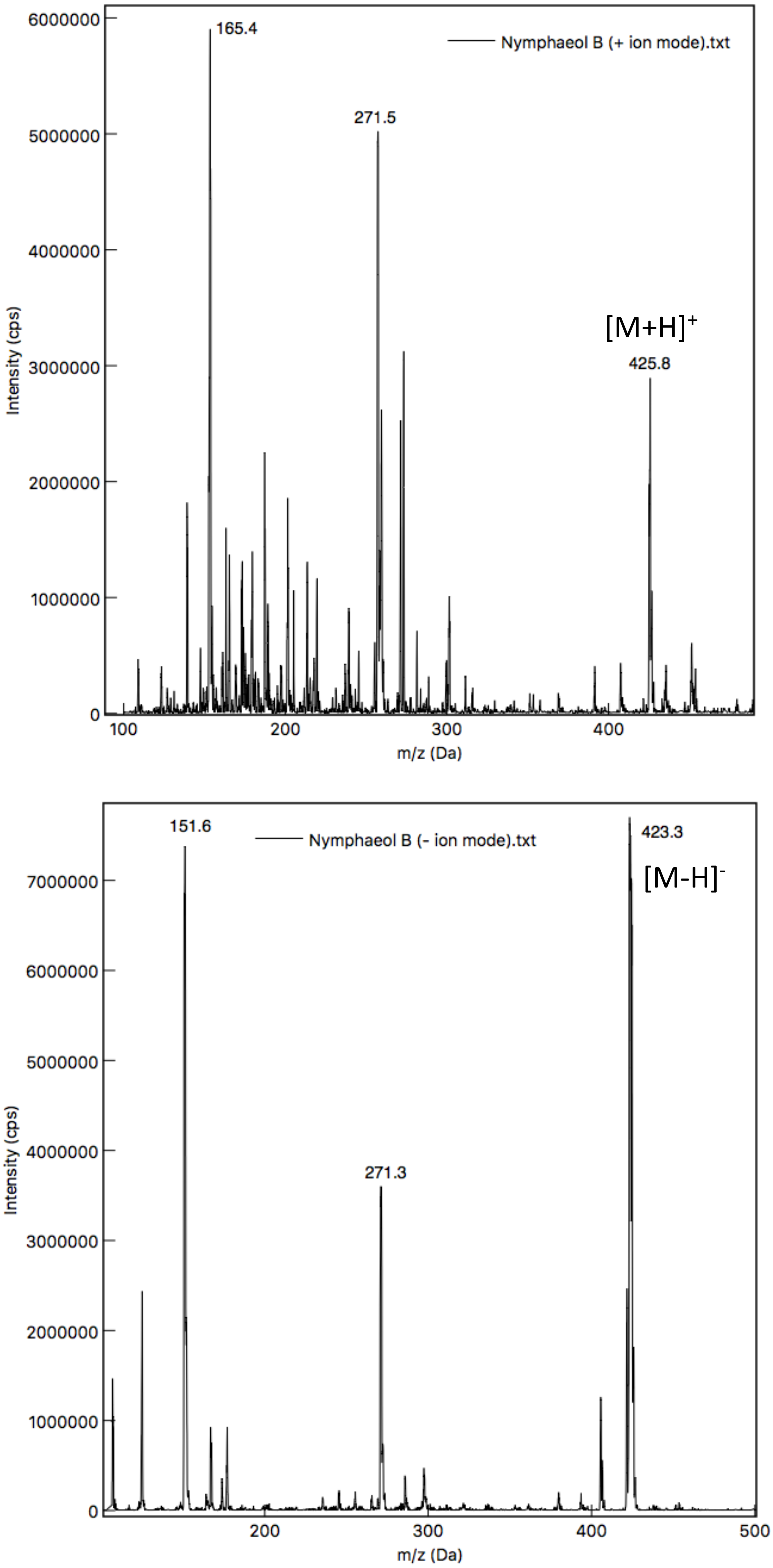

Figure S31. ESI-MS spectrum of **2** by UPLC-MS analysis.

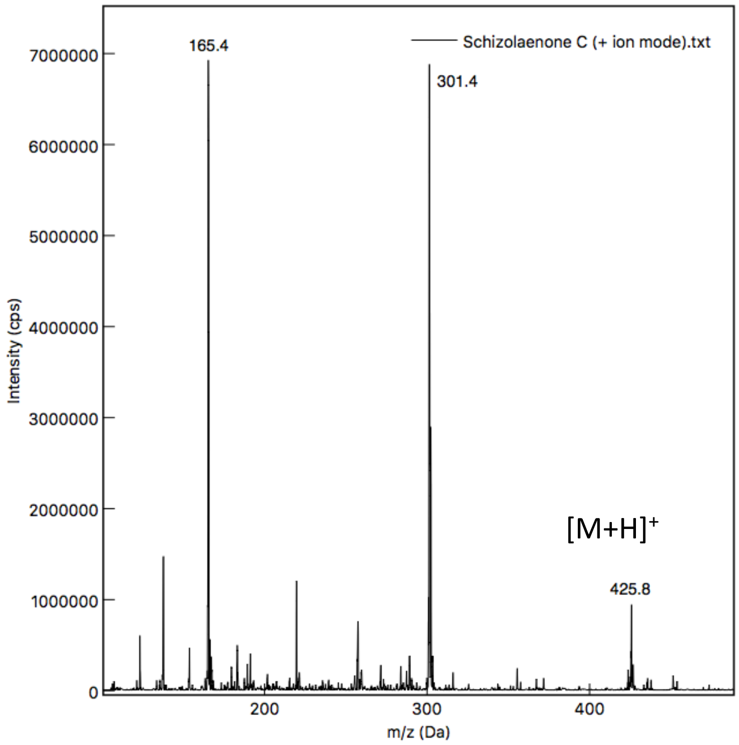

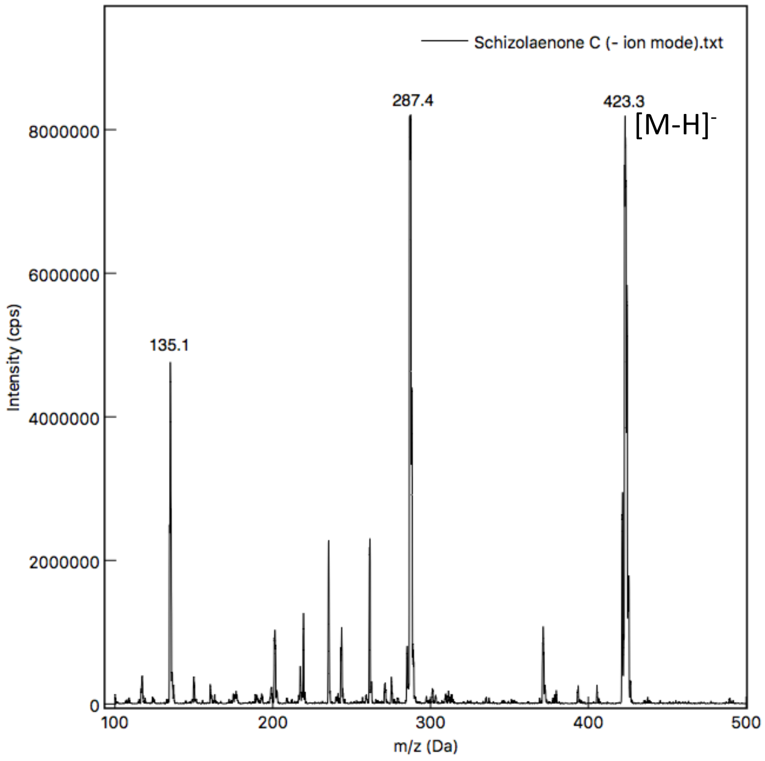

Figure S32. ESI-MS spectrum of **3** by UPLC-MS analysis.

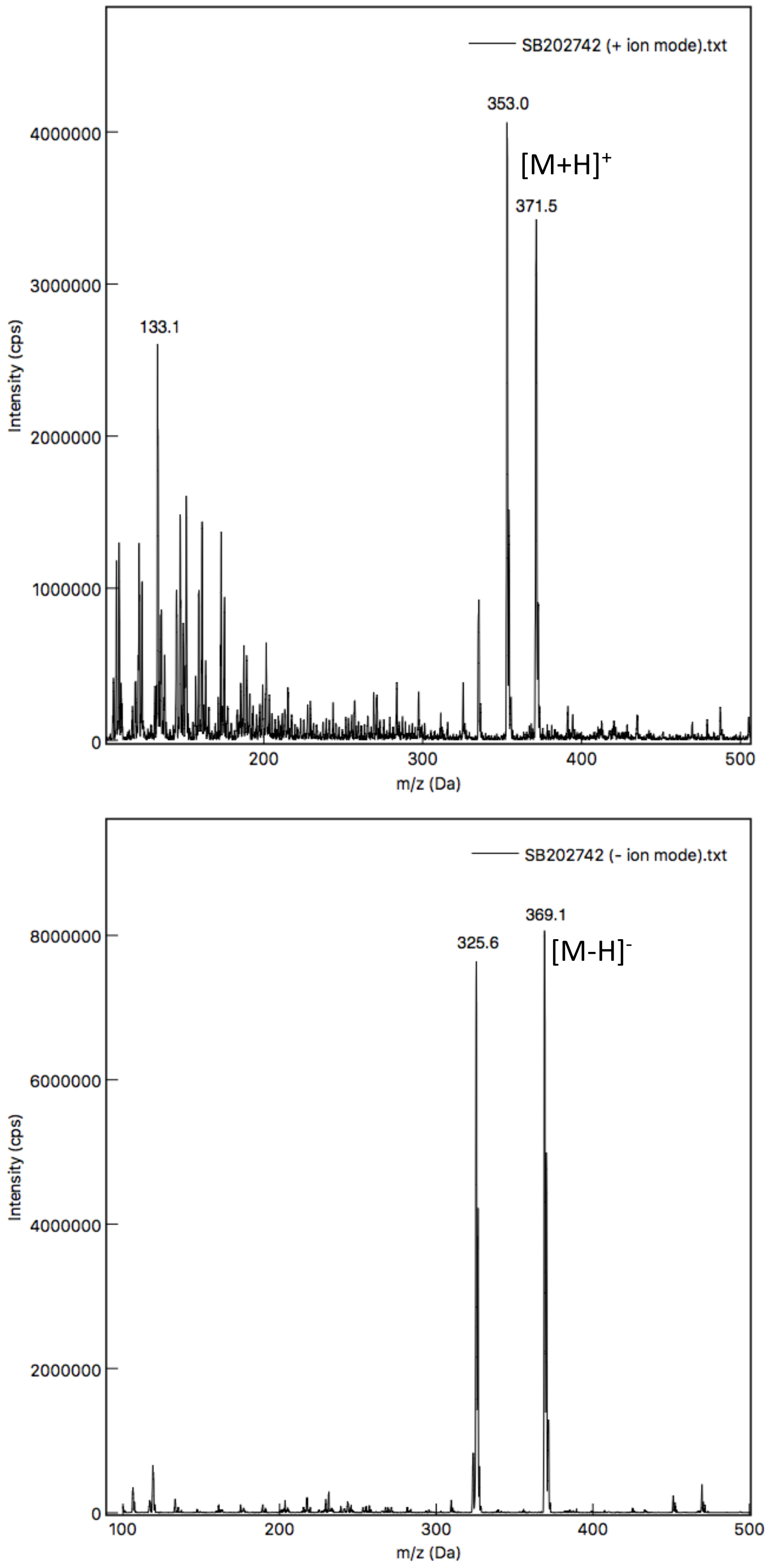

Figure S33. ESI-MS spectrum of compound **4** by UPLC-MS analysis.

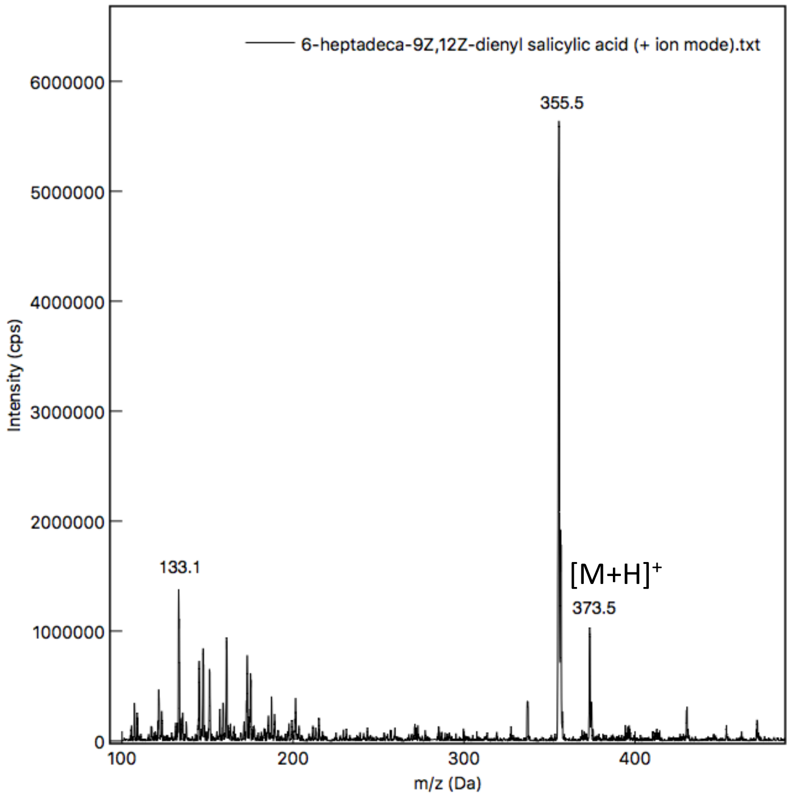

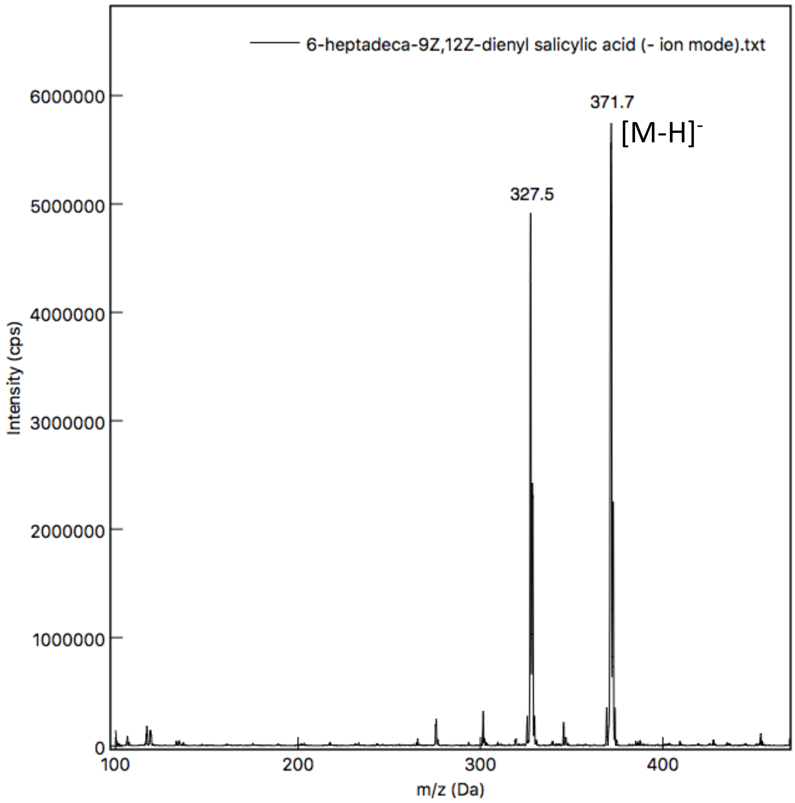

Figure S34. ESI-MS spectrum of **5** by UPLC-MS analysis.

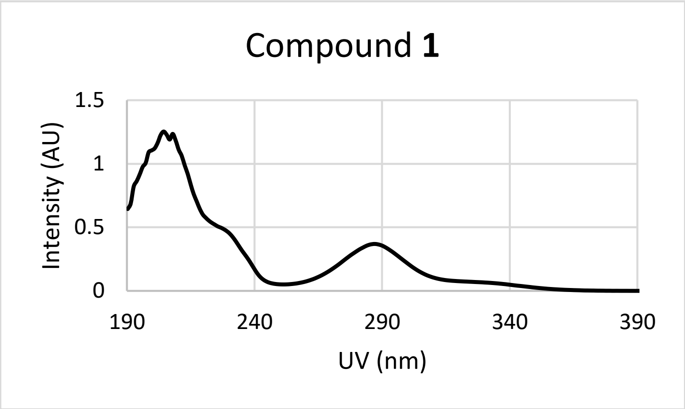

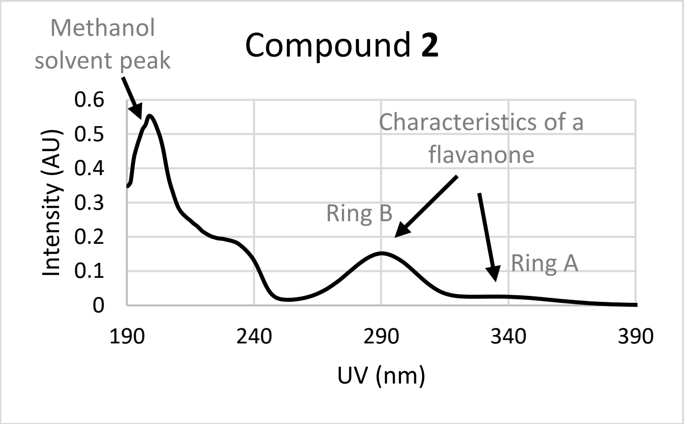

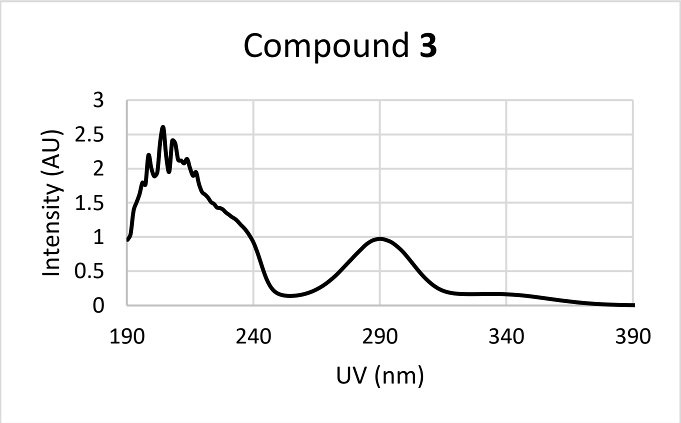

Figure S35. UV spectra of **1**, **2**, and **3** by UPLC-MS analysis.

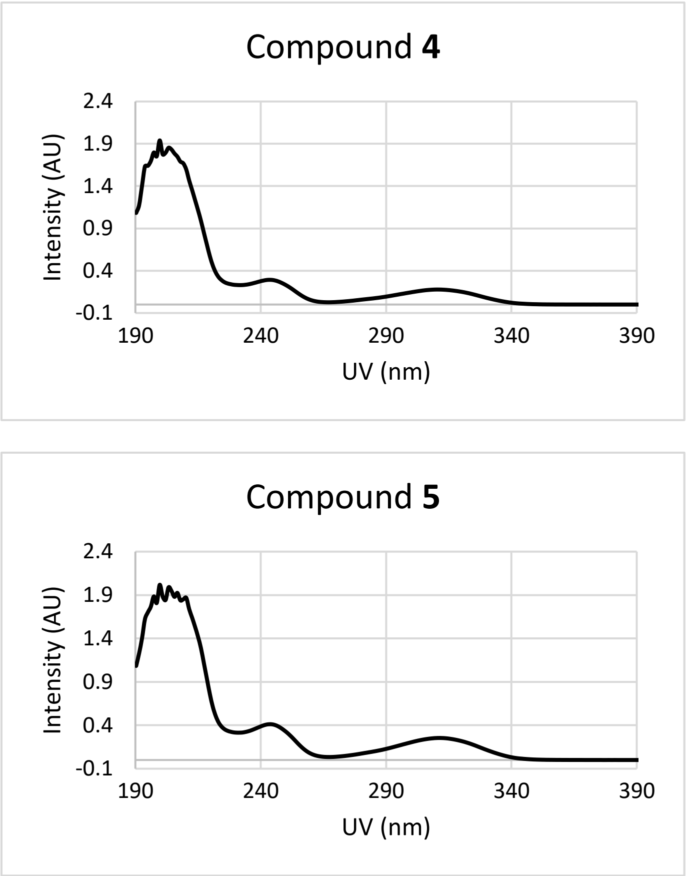

Figure S36. UV spectra of **4** and **5** by UPLC-MS analysis.

Table S1. Physicochemical properties of **1** – **5**.

Compound	**1**	**2**	**3**	**4**	**5**
Appearance			Yellow oil		
Molecular formula	$C_{56}H_{97}N_{11}O_{12}$	$C_{57}H_{99}N_{11}O_{12}$	$C_{57}H_{99}N_{11}O_{12}$	$C_{58}H_{101}N_{11}O_{12}$	$C_{59}H_{103}N_{11}O_{12}$
UV (MeOH) λ_{max} (log **ε)** (nm)	233, 289, 334	233, 287, 334	233, 290, 334	200, 243, 311	200, 243, 311
ESI-MS (*m*/*z*)	491.9 $[M–H]^-$ $C_{30}H_{35}O_6$	423.3 $[M–H]^-$ $C_{25}H_{27}O_6$	423.3 $[M–H]^-$ $C_{25}H_{27}O_6$	369.1 $[M–H]^-$ $C_{24}H_{33}O_3$	371.7 $[M–H]^-$ $C_{24}H_{35}O_3$

Table S2. Comparison of NMR chemical shifts of **1** with reported compounds, solophenol A [16] and nymphaeol C [4].

^{13}C	Compound 1 in CD_3OD		Solophenol A in acetone-d_6		Nymphaeol C in acetone-d_6	
	δ ^{13}C	δ^1H (J inHz)	δ ^{13}C	δ^1H (J in Hz)	δ ^{13}C	δ ^1H (J in Hz)
2	77.9	5.44, dd (3,13.5)	77.2	5.58, dd (2.7,13.3)	76.9	5.44, dd (3.2,12.4)
3	44.0	2.59, dd (3,17)<br>3.09, dd (13.5,17)	43.3	2.66, dd (2.7,17.3)<br>3.15, dd (13.3,17.3)	43.3	Eq 2.69, dd (3.2,17.2)<br>Ax 3.09, dd (12.4,17.2)
4	198.4		198.0		197	
5	163.0		163.0		161.8	
6	95.6	5.92, s	96.3	6.05, s	108.7	
7	166.1		164.9		164.4	
8	109.8		108.3		95	5.92, s
9	162.6		161.5		161.8	
10	103.2		103.2		102.7	
1'	130.0		130.1		129.5	
2'	128.3		127.6		127.1	
3'	144.5		144.1		143.6	
4'	146.5		145.6		145.1	
5'	113.7	6.72, d (8)	113.5	6.84, d (8.3)	113.2	6.91, d (8)
6'	118.7	6.88, d, (8)	118.6	7.01, d (8.3)	118.1	6.74, d (8)
1''	25.5	3.47, d	25.2	3.58, m	25.1	3.36
2''	124.8	5.11, t (6,6)	123.8	5.16, m	123.7	5.11
3''	135.8		135.4		135	
4′′	16.5	1.65, d (5)	16.3	1.69, s	16.3	1.74
5''	40.9	1.95, t (7,7)	40.4	1.97, m	40.2	2.02
6''	27.8	2.03, q (14,15)	27.4	2.05, m	27.2	2.02
7''	125.5	5.02, t (6,7)	124.3	5.16, m	124.6	5.11
8''	132.4		131.8		131.3	
9''	26.0	1.60, s	25.8	1.61, s	17.8	1.56
10''	17.9	1.54, s	17.7	1.56, s	25.7	1.64
1'''	22.0	3.21, d	22.3	3.22, m	21.5	3.36,
2'''	124.1	5.20, t (6,7)	125.0	5.06, m	123.3	5.11
3'''	131.7		131.2		130.7	
4'''	26.1	1.65, d (5)	25.9	1.61, s	17.8	1.8
5'''	17.998	1.75, s	17.8	1.58, s	25.7	1.74

s: singlet; d: doublet; dd: doublet of doublet; m: multiplet; Eq: equatorial; Ax: axial

Table S3. Comparison of NMR chemical shifts of **2** with reported compound, nymphaeol B [4].

^{13}C	**Compound 2 in CD_3OD**		**Nymphaeol-B in acetone- *d_6***	
	**δ ^{13}C**	**δ ^{1}H (*J* in Hz)**	**δ ^{13}C**	**δ ^{1}H (*J* in Hz)**
2	77.5	5.47, dd (3,13)	77	5.61, dd (3.2,12.6)
3	43.8	2.60, dd (3,18)	43.1	Eq 2.65 dd (3.2,17.2)
		3.11, dd (13,17)		Ax 3.17, dd (12.6,17.2)
4	198.2		197	
5	165.3		164.8	
6	97.4	5.87, s	96.6	5.97, s
7	169.2		167	
8	96.5	5.87, s	95.6	5.97, s
9	165.7		164.2	
10	103.2		102.8	
1′	129.9		129.4	
2′	128.4		127.3	
3′	144.6		143.7	
4′	146.6		145.2	
5′	113.7	6.72, d (8)	113.2	6.98, d (8)
6′	118.8	6.89, d, (8)	118.2	6.81, d (8)
1′′	25.5	4.48, d (7)	25.1	3.54
2′′	124.8	5.11, t (5,7)	123.8	5.10
3′′	135.8		135.2	
4′′	16.5	1.65, s	16.3	1.7
5′′	40.9	1.96, t (8,7)	40.3	2.00
6′′	27.8	2.03, q (7,14)	27.2	2.00
7′′	125.5	5.03, t (7,7)	124.7	5.10
8′′	132.4		131.4	
9′′	17.9	1.55, s	17.6	1.56
10′′	26.0	1.61, s	25.7	1.62

s: singlet; d: doublet; dd: doublet of doublet; m: multiplet; t: triplet; q: quartet; Eq: equatorial; Ax: axial

Table S4. Comparison of NMR chemical shifts of **3** with reported compound, schizolaenone C [17].

^{13}C	Compound 3 in CD_3OD		Schizolaenone C in CD_3OD	
	**δ ^{13}C**	**δ ^1H (*J* in Hertz)**	**δ ^{13}C**	**δ ^1H (*J* in Hertz)**
2	80.6	5.25, dd (3,12.5)	80.4	5.24, dd (3.2, 12.8)
3	44.4	2.68, dd (3,17)	44.2	Eq 2.68, dd (3.2, 16.8)
		3.05, dd (12.5,17)		Ax 3.05, dd (12.8, 16.8)
4	197.9		197.8	
5	162.6		162.4	
6	109.9		109.7	
7	166.4		165.9	
8	95.6	5.94, s	95.4	5.94, s
9	162.7		162.5	
10	103.3		103.2	
1′	132.2		131.9	
2′	119.4	6.79, s	119.2	6.91, s
3′	147.0		146.8	
4′	116.4	6.79, s	116.2	6.78, s
5′	146.7		146.5	
6′	114.8	6.91, s	114.7	6.78, s
1′′	21.9	3.21, d (8)	21.8	3.21, d (7.2)
2′′	124.2	5.19, t (6,7)	123.9	5.19, m
3′′	135.4		135.2	
4′′	16.3	1.75, s	16.2	1.74, s
5′′	41.1	1.95, t (8,7)	40.9	2.04, m
6′′	27.9	2.05, q (6,8)	27.7	1.94, m
7′′	125.6	5.06, t (7,7)	125.4	5.06, m
8′′	132.1		132	
9′′	26.0	1.62, s	25.8	1.62, s
10′′	17.8	1.56, s	17.7	1.56, s

s: singlet; d: doublet; dd: doublet of doublet; m: multiplet; t: triplet; q: quartet; Eq: equatorial; Ax: axial

Table S5. Comparison of NMR chemical shifts of **4** with reported compound, SB-202742 [18].

	Compound 4 in $CDCl_3$			SB-202742		
^{13}C	δ ^{13}C	δ ^1H	Multiplicity (*J* in Hertz)	δ ^{13}C	δ ^1H	Multiplicity (*J* in Hertz)
**1**	110.7			118.6		
**2**	163.8			162.3		
**3**	116.1	6.85	d (7.5)	115.2	6.65	dd (7.9,1.0)
**4**	135.6	7.34	t (7.5)	132.4	7.11	dd (7.7,7.9)
**5**	122.9	6.75	d (7.5)	122.5	6.61	dd (7.7,1.0)
**6**	147.9			147.5		
**7**	175.8			176.5		
**1′**	36.7	2.96	t (8.0)	36.4	3.02	t (7.7)
**2′**	32.2	1.58	qu	33.3	1.58	br
**3′**	29.5	1.32	br	30.1	1.31	br
**4′**	30.0			30.8		
**5′**	29.9			30.6	1.28	br
**6′**	29.6			30.4	1.26	br
**7′**	20.8	2.05	br	21.5	2.06	br
**8′**	132.2	5.33	br	132.7	5.36	br
**9′**	130.6			131.2		
**10′**	25.8	2.79	t (6.5)	26.4	2.79	br
**11′**	128.5	5.33	br	129.2	5.31	br
**12′**	128.5			129.2		
**13′**	25.8	2.79	t (7.0)	26.0	2.79	br
**14′**	127.9	5.33	br	128.2	5.30	br
**15′**	127.3			128.2	5.27	br
**16′**	27.5	2.05	br	28.2	2.07	br
**17′**	14.49	0.95	t (6.5)	14.7	0.95	br

s: singlet; d: doublet; dd: doublet of doublet; m: multiplet; t: triplet; q: quartet; qu: quintet; br: broad

Table S6. Comparison of NMR chemical shifts of **5** with reported compound, 6-heptadeca-9*Z*,12*Z*-dienyl salicylic acid [19].

^{13}C	Compound 5 in $CDCl_3$			6-[(9Z,12Z)-heptadeca dienyl]salicylic acid		
	δ ^{13}C	δ ^{1}H	Multiplicity (*J* in Hertz)	δ ^{13}C	Δ ^{1}H	Multiplicity (*J* in Hertz)
**1**	111.7			110.68		
**2**	163.4			163.48		
**3**	115.6	6.79	d (7.5)	115.79	6.85	dd (7.9,1.0)
**4**	134.5	7.26	t (7.5)	135.21	7.34	t (7.9)
**5**	122.5	6.69	d (7.5)	122.67	6.76	dd (7.9,1.0)
**6**	147.3			147.61		
**7**	174.1			175.64		
**1′**	36.6	2.92	t (8.0)	36.41	2.97	t (7.6)
**2′**	32.3	1.54	qu (7.5)	31.97	1.60	qu (7.6)
**3′**	31.7			31.50		
**4′**	30.0	1.28	br	29.74	1.31	br
**5′**	29.9			29.64		
**6′**	29.6			29.37		
**7′**	29.5			29.33		
**8′**	27.4	2.01	q (7.0)	27.18	2.04	br
**9′**	130.4	5.32	br	130.19	5.35	br
**10′**	130.3			130.19		
**11′**	25.8	2.74	t (6.5)	25.61	2.77	t (5.6)
**12′**	128.2	5.32	br	127.94	5.35	br
**13′**	128.1			127.94		
**14′**	27.4	2.01	q (7.0)	27.39	2.04	br
**15′**	29.5	1.28	br	29.24	1.31	br
**16′**	22.8			22.46		
**17′**	14.2	0.85	t (6.5)	14.05	0.88	t (7.0)
**OH**					10.84	t

s: singlet; d: doublet; dd: doublet of doublet; m: multiplet; t: triplet; q: quartet; qu: quintet; br: broad
